# A narrative review of the impact of the transition to ICD-10 and ICD-10-CM/PCS

**DOI:** 10.1093/jamiaopen/ooz066

**Published:** 2019-12-26

**Authors:** Sheila V Kusnoor, Mallory N Blasingame, Annette M Williams, Spencer J DesAutels, Jing Su, Nunzia Bettinsoli Giuse

**Affiliations:** 1 Center for Knowledge Management, Strategy and Innovation, Vanderbilt University Medical Center, Nashville, Tennessee, USA; 2 Department of Biomedical Informatics, Vanderbilt University School of Medicine, Vanderbilt University Medical Center, Nashville, Tennessee, USA; 3 Department of Medicine, Vanderbilt University Medical Center, Nashville, Tennessee, USA

**Keywords:** International Classification of Diseases, clinical coding, population surveillance, diagnosis codes, claims

## Abstract

**Objectives:**

The United States transitioned to the tenth version of the International Classification of Diseases (ICD) system (ICD-10) for mortality coding in 1999 and to the International Classification of Diseases, Clinical Modification and Procedure Coding System (ICD-10-CM/PCS) on October 1, 2015. The purpose of this study was to conduct a narrative literature review to better understand the impact of the implementation of ICD-10/ICD-10-CM/PCS.

**Materials and Methods:**

We searched English-language articles in PubMed, Web of Science, and Business Source Complete and reviewed websites of relevant professional associations, government agencies, research groups, and ICD-10 news aggregators to identify literature on the impact of the ICD-10/ICD-10-CM/PCS transition. We used Google to search for additional gray literature and used handsearching of the references of the most on-target articles to help ensure comprehensiveness.

**Results:**

Impact areas reported in the literature include: productivity and staffing, costs, reimbursement, coding accuracy, mapping between ICD versions, morbidity and mortality surveillance, and patient care. With the exception of morbidity and mortality surveillance, quantitative studies describing the actual impact of the ICD-10/ICD-10-CM/PCS implementation were limited and much of the literature was based on the ICD-10-CM/PCS transition rather than the earlier conversion to ICD-10 for mortality coding.

**Discussion:**

This study revealed several gaps in the literature that limit the ability to draw reliable conclusions about the overall impact, positive or negative, of moving to ICD-10/ICD-10-CM/PCS in the United States.

**Conclusion:**

These knowledge gaps present an opportunity for future research and knowledge sharing and will be important to consider when planning for ICD-11.

## INTRODUCTION

The International Classification of Diseases (ICD) system was developed by the World Health Organization (WHO) as a standardized method for tracking diseases across populations. WHO has made major revisions in content and structure over the past several decades, adapting to new scientific understanding of disease and new structures of organizing ICD codes to accommodate improved use and extensibility.^1^ The tenth revision of ICD (ICD-10) greatly expanded the number of categories from nearly 5000 to approximately 8000 and is currently used in more than 100 countries.^2^ Modifications to ICD, such as the ICD-10-Clinical Modification (ICD-10-CM), ICD-10-Procedure Coding System (ICD-10-PCS), and country-specific modifications, extend the specificity of ICD-10 to new needs, such as reimbursement in addition to surveillance.^1^ The United States adopted ICD-10 for mortality reporting in 1999, and ICD-10-CM and ICD-10-PCS for morbidity surveillance and reimbursement on October 1, 2015. These changes in scope and arrangement provide both benefits and drawbacks. The scale of the revisions and modifications requires organizations worldwide to make significant changes to processes and information systems that use ICD codes, especially in the modern era of health information technology and widespread adoption of the ICD classification.

The eleventh revision of ICD (ICD-11), reflecting updates based on medical, scientific, and technological advances, was presented at the World Health Assembly of WHO on May 25, 2019, and is expected to become effective in 2022.^3^ ICD-11 offers greater detail, increasing the number of unique codes for injuries, diseases, and causes of death from approximately 14 400 codes in ICD-10 to nearly 55 000 codes in ICD-11.^4^ A key distinguishing feature of ICD-11 is also that it is fully electronic, which is anticipated to facilitate its ease of use and implementation.^4^ As nations begin to prepare for ICD-11, it is helpful to reflect on the US experience of the ICD-9/ICD-9-CM to ICD-10/ICD-10-CM/PCS transition. Thoroughly understanding the impact of these transitions on patients, providers, and healthcare systems may allow the many varied stakeholders to better prepare for future conversions and other similar transformational events.

## OBJECTIVE

The purpose of this narrative review was to identify and describe areas impacted by the implementation of ICD-10 and the US adoption of ICD-10-CM/PCS, including the costs, benefits, and challenges.

## MATERIALS AND METHODS

We conducted a comprehensive review of the published and gray literature regarding the impact of the transition to ICD-10 and ICD-10-CM/PCS. Given the broad scope of the topic, a systematic review of the literature would not be appropriate or feasible. Therefore, we conducted a narrative, comprehensive literature review to describe the state of the literature evaluating the impact of the implementation.

We searched articles indexed in the electronic databases PubMed, Web of Science, and Business Source Complete by May 10, 2019, using comprehensive search strategies that included terms related to ICD-10, transitioning, and potential impact (Supplementary File S1). The searches were refined through multiple rounds of testing and informed by a preliminary review of the literature,^5–9^ including the projected impact of the transition, and feedback from the National Committee on Vital and Health Statistics (NCVHS). We also searched websites of relevant professional associations, government agencies, research groups, and ICD-10 news aggregators to identify additional literature and conducted gray literature searches using Google to capture white papers, conference proceedings, and presentations. Additional citations were located by handsearching the references cited by the most on-target articles.

Articles were deemed relevant if they addressed the impact of the transition, including the benefits, costs, and challenges, and were written in English. We focused on studies that assessed the actual impact of the transition rather than solely providing information about hypothetical impact or projections. To ensure interrater reliability of the screening process for articles retrieved through the electronic database searches, three reviewers independently screened a set of 100 title/abstracts from the PubMed search retrieval and then convened as a group to adjudicate final decisions. With interrater reliability at 85%, team members performed independent, single-reviewer screening of the remainder of the citations. Gray literature sources were also reviewed through independent, single-reviewer screening. A synthesis of the most salient literature regarding areas of impact that were identified through the review is provided below. See Supplementary File S2 for information about additional studies identified addressing these impact areas and Supplementary File S3 for articles addressing other issues related to the transition that were beyond the scope of the narrative review.

## RESULTS

### Productivity and staffing

Reports in the literature describe a change in coder and/or provider productivity and staffing for some organizations after the ICD-10-CM/PCS implementation. For example, a CIOX/University of Pittsburgh analysis of average ICD-10 coding time of 157 248 inpatient records found that while the average coding time improved from October 2015 (43.7 min/record) to February 2016 (40.4 min/record), rates were still 20% below productivity levels when compared to a previous ICD-9 CIOX dataset of 84 627 records and 50% below American Health Information Management Association (AHIMA) recommended ICD-9 productivity levels.^10^ The study was extended to include an additional 165 864 records from March 2016 to July 2016.^11^ A continued improvement in ICD-10 coding productivity was observed, with an average coding time of 37.45 minutes in July 2016.^11^ A retrospective study of a mid-sized—10 physicians, 1 coder, 6 administrators—ophthalmology practice similarly found that coder efficiency was reduced 4 months after the October 2015 ICD-10 implementation, but it had returned to baseline in the following 8 months.^12^ The same study found no change in clinical volume when comparing the periods 12 months before and after ICD-10 implementation, which was attributed to the practice’s use of a certified coding team, which decreases the amount of physician time needed for coding.^12^ Physicians Foundation’s April–June 2016 biennial survey of American physicians registered with the American Medical Association also found that approximately 43% of 17 236 physician respondents reported that ICD-10 detracted from practice efficiency, while 6% reported that it improved efficiency.^13^

A Healthcare Billing and Management Association member survey,^14^ conducted in February 2016, addressed changes in staffing. The survey included 38 billing companies as respondents and found that 5 companies hired additional coders, 7 companies outsourced coding, 9 companies added automated coding tools, and 17 companies made no changes.^14^

### Costs

Conversion to ICD-10-CM/PCS resulted in costs for many organizations due to the need for training, software, and testing. Reports of the costs spent due to ICD-10-CM/PCS implementation varied in the literature. A December 2015 Navicure survey of 360 physician practices, which asked respondents to indicate the amount their organization spent on software updates and training, found 9% reported no costs, approximately 50% of respondents reported spending less than $10 000, 14% spent from $10 000–$49 000, 2% spent $50 000–$99 999, 1% spent $100 000–$199 999, and 2% spent $200 000 or more.^15^ The majority of respondents (60%) were from practices with 1–10 providers. The study did not address differences in costs based on organization size or scope of training. A study from a single academic surgery department reported that $390 000 was spent on provider training, which included efforts to improve clinical documentation as a key component.^16^ A December 2014 to January 2015 survey by the Professional Association of Health Care Office Management (PAHCOM) assessed costs due to ICD-10 conversion for small practices (≤6 providers), including costs for ICD-10 manuals and documentation, software upgrades and testing, training, and superbill conversion to ICD-10.^17^ The study included 276 small practices and found that the average ICD-10-related expenditures were $3430 per provider.^17^

Implementation of ICD-10-CM/PCS was twice delayed in the United States, from an original target date in 2013 to the final implementation in 2015. The Protecting Access to Medicare Act of 2014, which delayed the ICD-10-CM/PCS compliance date from 2014 to 2015, was anticipated to result in additional costs to some organizations. In an Emergency Summit convened by the Workgroup for Electronic Data Interchange (WEDI), which was attended by approximately 200 individuals representing payers, providers, vendors, and government agencies, organizations estimated the delay would cost them up to an additional 5–10 million dollars, including incremental costs to maintain two application code bases and remove date triggers to use ICD-10 codes.^18^ A 2014 Wall Street Journal blog reports that the 2014 delay was expected to increase training costs at St. Luke’s Health System, which at the time of the report included 10 hospitals, from $300 000 to $450 000 due to the need for extending physician training by an additional year.^19^

### Reimbursement

The reported impact of ICD-10-CM/PCS conversion on reimbursement varied in the literature. A December 2015 Navicure survey that included 360 physician practices^15^ found that monthly revenue was not impacted after the October 2015 conversion to ICD-10 for 60% of respondents; however, 34% of respondents reported a revenue decrease of up to 20%, 5% of respondents reported a revenue decrease of 21–40%, and 1% reported a revenue decrease of 41–60%. In a 2016 Physicians Foundation survey, 17 236 physicians self-reported the overall impact of ICD-10 on revenues for their practices within predefined categories: 24.1% of respondents reported that ICD-10 detracted from revenues, 6% reported improved revenues, and 69.9% indicated it had little or no impact on revenues.^13^ A retrospective study of an academic ophthalmology practice found that per 100 visits, coding-related denials, charges denied, and percentage of charges denied nearly doubled; however, there was no change in total revenue based on an analysis of data 12 months before and after the October 2015 ICD-10 conversion.^12^ The transaction-processing firm Change Healthcare reported that 5% of their clients had a time to bill increase greater than 5 days based on a November 2015 survey; however, for two-thirds of their clients, time to bill did not change.^20^

### Coding accuracy

A few studies suggest coding accuracy may have been impacted as a result of the ICD-10-CM/PCS transition. In the February 2016 Healthcare Billing and Management Association (HBMA) survey of 38 billing companies,^14^ 14 companies reported no change in coding accuracy, 11 companies reported increased accuracy, 7 companies reported decreased accuracy, and 2 companies reported significantly increased coding errors.

In a qualitative study conducted by the AHIMA Foundation,^21^ phone interviews were conducted with a random sample of coding professionals in the AHIMA member database. Of 156 respondents, 38% of respondents (*n* = 60) indicated perceived changes in accuracy following ICD-10 implementation, with 18 respondents reporting an average perceived increase in accuracy of 25% and 42 participants indicating an average perceived decrease in accuracy of 13%.^21^ These data are based on respondents’ perceived accuracy levels as self-reported in the phone interviews; specific details of how accuracy levels were determined were not reported.

Stitcher and Lawrence of Horizon Consulting, an information technology consulting and implementation services firm, presented an analysis of post-ICD-10 implementation coding based on over 30 000 records with discharge dates from October 1, 2015, to June 30, 2016, from the Maryland Health Services Cost Review Commission public use discharge dataset.^22^ Upon manual review, changes in coding were recommended for 16% of cases that would have affected severity of illness and/or all patient refined diagnosis-related groups. The average change in case mix points per case was 0.297 in ICD-9 compared with 0.341 in ICD-10, with thoracic surgery, neurosurgery, and ventilator support having the largest changes.^22^ The authors did not conduct a statistical significance analysis.

### Mapping

One of the challenges resulting from ICD-10 and ICD-10-CM/PCS implementation was mapping between versions. For example, Boyd et al.^23^ evaluated the complexity of ICD-9-CM to ICD-10-CM mappings from 24 008 billing diagnoses in a 2010 Illinois Medicaid dataset and found that 36% of ICD-9-CM code mappings to ICD-10-CM were convoluted (ie, ambiguous or complex), possibly resulting in more work on the part of the coder as well as discontinuities in disease reporting. A second study assessing the complexity in mapping between ICD-9-CM-Vol3 and ICD-10-PCS using procedure codes for 3290 patients from the same 2010 Illinois dataset found that 55% were convoluted, 40% were simple, and 5% had no mapping.^24^ The complexity in mapping creates an ongoing factor to be considered in research spanning ICD-9-CM and ICD-10-CM/PCS data, as was addressed by the Healthcare Cost Utilization Program’s recommendations for mapping strategies.^25^

### Morbidity surveillance

Several studies have assessed the impact of the conversion to ICD-10-CM on morbidity surveillance, with some studies reporting disruptions in disease tracking due to changes in the coding system. A study using inpatient Medicare data from 2012 to 2015 found sudden changes in the frequency of certain diseases after the adoption of ICD-10-CM in the fourth quarter of 2015, with discontinuities ranging from −8.9% (cardiac arrhythmias) to +10.9% (psychoses).^26^ Another study compared ICD-9-CM and ICD-10-CM codes for 34 chronic conditions using data from fiscal years 2014–2016.^27^ In a random sample of 1 million patients in the Veterans Affairs health system, the authors found that diagnoses were largely consistent across the transition period, with some notable exceptions (eg, higher odds of Alzheimer’s disease and spinal cord injury measurement; lower odds of HIV/AIDS and arthritis measurement).

However, other studies have seen that ICD-10-CM conversion had a minimal impact on morbidity surveillance. For example, Kelley et al.^28^ evaluated the impact of the 2015 ICD-9 to ICD-10 transition codes on the ability to define populations with serious illness. The study compared data from the National Health and Aging Trends Study, a longitudinal survey of older adults in the United States, over 6-month follow-up periods in 2014 (ICD-9) and 2016 (ICD-10) to test the hypothesis that Medicare costs, healthcare utilization, and mortality would be similar between the two groups. The study found no significant differences between the samples.

### Mortality surveillance

Some studies suggest that ICD-10 implementation may have impacted mortality surveillance. For example, Anderson et al.^2^ estimated the effects of the ICD-10 transition on cause of death data in the United States and found that the rankings of the top 10 causes of death were affected. The study used a sample of 80% (1,852,671/2, 314, 690) of all resident deaths in the United States in 1996 based on death certificates from all 50 states and Washington, D.C. While the top five causes of death in 1996 remained the same in ICD-10 coding, the conditions ranked sixth and seventh (influenza & pneumonia and diabetes) changed places, and Alzheimer’s disease entered the top 10 at the eighth position, resulting in shifting down of two causes and in chronic liver disease and cirrhosis dropping out of the top 10 altogether in this preliminary estimate.^2^ Another study attributed the change in the leading cause of death in Puerto Rico from heart disease to cancer in 2002 to the ICD-10 transition, which resulted in an increase in classification of hypertensive disease deaths and a decrease in heart disease deaths.^29^

Similarly, other studies report an impact on mortality surveillance for specific conditions. The 1999 transition to ICD-10 in the United States was reported to have impacted classification of maternal deaths, with a 13% increase in maternal deaths seen from 1998 to 1999; this change was attributed in part to increased coding of “indirect” deaths as maternal deaths and other modifications in coding rules.^30^ When taking the comparability ratio into account, the difference was not statistically significant.^30^

### Patient care

A few physician surveys have assessed the impact of the October 2015 ICD-10 transition on patient care. A November 2015 poll from the physician social media network SERMO (*N* = 1249 physicians) found that two-thirds of respondents indicated that ICD-10 had detracted from their time with patients; however, this was an improvement from the previous month’s poll, where 86% of respondents reported a negative impact on patient care.^31^ In a 2016 Physicians Foundations survey that included 17 236 physician respondents, 27.9% reported that ICD-10 detracted from patient care, and 5% reported that it improved patient care in their practice; however, patient care was not defined in the report.^13^

## DISCUSSION

This narrative review highlights the state of the literature on the impact of the ICD-10/ICD-10-CM/PCS transition, including the effects on productivity and staffing, costs, reimbursement, coding accuracy, mapping between ICD versions, morbidity and mortality surveillance, and patient care. Much of the reported literature was based on the ICD-10-CM/PCS transition rather than the earlier transition to ICD-10. Few articles addressed the longer-term impact of the implementation of ICD-10/ICD-10-CM/PCS, and, with the exception of the studies on disease tracking and mapping between ICD versions, much of the evidence was qualitative. Limited data were available on the experience of large healthcare centers. Overall, our review was unable to establish significant evidence of harm or benefit based on the available literature.

Several knowledge gaps were identified from the literature review including: the costs of the ICD-10/ICD-10-CM/PCS transition for organizations of various sizes, how coding accuracy was impacted based on a comparison of ICD-9/ICD-9-CM data with ICD-10/ICD-10-CM, and whether the increased specificity of codes led to improved reimbursement. While physician survey data indicates that patient care may have been impacted by ICD-10-CM/PCS implementation, additional studies are needed to confirm these findings and evaluate the impact on other aspects of care. A potential impact on patient care is not unexpected given the time needed for learning and using a new system.

Prior to the 2015 implementation, some stakeholders predicted that ICD-10-CM/PCS implementation would have worrying financial implications for the US healthcare system.^32^^,^^33^ The reported findings on the costs of the 2015 transition are largely based on surveys, where respondents may or may not have been equipped with real data. Insufficient data were available to extrapolate how the cost findings apply to different environments. Given this gap in the literature, it was not possible to evaluate the full financial impact in comparison with highly cited projected costs.^34^

The ICD-10-CM/PCS implementation appears to have resulted in discontinuities in data tracked over the transition period, across research, epidemiologic, and clinical settings. However, aside from highlighting the need to account for transition effects when analyzing such data, the full implications of this finding are unclear, as not all conditions were affected, and the source of the discontinuities—whether due to mapping, coding errors, or changes (positive or negative) in the codes themselves—was generally not established. Methodologic variability also limited further synthesis. However, given the evidence for disruptions in morbidity and mortality surveillance for specific conditions with the ICD-10 transition, it will be important to identify disease areas/conditions that are most likely to be impacted by the change in codes with ICD-11 and identify strategies for minimizing the confounding role they may play when using or analyzing data coded with different ICD versions.

### Limitations

Use of a single reviewer per document for initial screening for eligibility may have resulted in the inadvertent exclusion of relevant articles during the citation screening process; however, we carefully analyzed interrater agreement prior to commencing single review. Interrater variance was also mitigated by handsearching of cited references from highly relevant articles. Quality assessment (including risk of bias and strength of the evidence) was not performed, as the identified study types did not lend themselves to this type of formal assessment.

## CONCLUSION

This narrative review revealed significant gaps in the literature, limiting the ability to draw reliable conclusions regarding the impact, positive or negative, of moving to ICD-10 coding in the United States and, by extension, the potential impact of a future transition to ICD-11. These knowledge gaps present an opportunity for future research and knowledge sharing, which could help guide decisions about the upcoming transition to ICD-11. The current state of the literature has the potential to be misleading in that it may not represent the full breadth of implementation-related harms and benefits experienced throughout the healthcare system, as a preponderance of retrieved studies used qualitative methods, which make the data more subjective in interpretation. Additionally, there was an overall lack of reporting on many key outcomes, especially after the immediate ICD-10-CM/PCS post-implementation period. For example, limited data were available on the costs of the transition, particularly the costs experienced by large physician practices and organizations. The costs reported by smaller organizations in the literature are likely not generalizable for larger practices. Additional quantitative studies are needed to understand the true costs and benefits of the transition, including the effects of the frustrations, delays, and need for substantial training, and the degree to which morbidity coding was enhanced. An evaluation plan for how the impact of the transition to ICD-11 will be addressed should be defined in advance to ensure that quality data are collected and reported.

## AUTHOR CONTRIBUTIONS

All authors made substantial contributions to the conception and design of the review, or the acquisition, analysis, or interpretation of data for the work. All authors contributed to drafting the manuscript and to critically revising the text for important intellectual content. All authors provided final approval of the version to be published and agree to be accountable for all aspects of the work in ensuring that questions related to the accuracy or integrity of any part of the work are appropriately investigated and resolved.

## Supplementary Material

ooz066_Supplementary_Data

## References

[1] Hirsch JA, Nicola G, McGinty G, et al ICD-10: history and context. AJNR Am J Neuroradiol 2016; 37 (4): 596–9. 26822730 10.3174/ajnr.A4696 PMC7960170

[2] Anderson RN, Miniño AM, Hoyert DL, et al Comparability of cause of death between ICD–9 and ICD–10: preliminary estimates. Natl Vital Stat Rep 2001; 49 (2): 1. 11381674

[3] World Health Organization (WHO). ICD-11: classifying disease to map the way we live and die. 2018 https://www.who.int/news-room/spotlight/international-classification-of-diseases Accessed October 16, 2019.

[4] World Health Organization (WHO). ICD-11 implementation or transition guide. 2019 https://icd.who.int/docs/ICD-11%20Implementation%20or%20Transition%20Guide_v105.pdf Accessed October 16, 2019.

[5] Libicki M, Brahmakulam I. The costs and benefits of moving to the ICD-10 code sets. Rand Science and Technology Technical Report prepared for the Department of Health and Human Services. 2004 https://www.rand.org/content/dam/rand/pubs/technical_reports/2004/RAND_TR132.pdf. Accessed October 16, 2019.

[6] Bowman S. A look back on the ICD-10 transition: crisis averted or imaginary? J AHIMA 2016; 87 (8): 24–31. 29425006

[7] Butler R. The ICD-10 general equivalence mappings. Bridging the translation gap from ICD-9. J AHIMA 2007; 78 (9): 84–5. 17987945

[8] Foley MM. DRG grouping and ICD-10-CM/PCS. J AHIMA 2015; 86 (7): 56–9. 26642625

[9] Stanfill MH, Hsieh KL, Beal K, et al Preparing for ICD-10-CM/PCS implementation: impact on productivity and quality. Perspect Health Inf Manag 2014; 11: 1f. PMC4142514 25214823

[10] Watzlaf V, Nemchik S, Hoerner M, et al ICD-10 Coding productivity study highlights emerging standards. J AHIMA 2016; 87 (8): 44–7. 29425014

[11] Alakrawi Z, Watzlaf V, Nemchik S, et al New study illuminates the ongoing road to ICD-10 productivity and optimization. J AHIMA 2017; 88 (3): 40–5. 29412544

[12] Hellman JB, Lim MC, Leung KY, et al The impact of conversion to International Classification of Diseases, 10th revision (ICD-10) on an academic ophthalmology practice. Clin Opthalmol 2018; 12: 949–56. 10.2147/OPTH.S161742 PMC5965384 29849450

[13] The Physicians Foundation. 2016 Survey of America’s Physician’s Practice Patterns & Perspectives. 2016. https://physiciansfoundation.org/wp-content/uploads/2018/01/Biennial_Physician_Survey_2016.pdf Accessed October 16, 2019.

[14] Louie H. Latest results: HBMA’s ICD-10 benchmark survey: three rev cycle companies shuttered. 2016 https://www.icd10monitor.com/latest-results-hbma-s-icd-10-benchmark-survey-three-rev-cycle-companies-shuttered Accessed October 16, 2019.

[15] Navicure. Healthcare organization post ICD-10 implementation survey: key survey findings. 2016 https://web.archive.org/web/20180219123237/http:/info.navicure.com/rs/669-OIJ-380/images/Navicure-Post-ICD-10-Survey_Final.pdf Accessed October 16, 2019.

[16] Reyes C, Greenbaum A, Porto C, et al Implementation of a clinical documentation improvement curriculum improves quality metrics and hospital charges in an academic surgery department. J Am Coll Surg 2017; 224 (3): 301–9. 27919741 10.1016/j.jamcollsurg.2016.11.010

[17] Blanchette K, Averill R, Bowman S. Survey of ICD-10 implementation costs in small physician offices. J AHIMA 2015 https://journal.ahima.org/wp-content/uploads/2015/02/Week-2_PAHCOM-Survey-Results.final_POST.pdf. Accessed October 16, 2019.

[18] Workgroup for Electronic Data Interchange (WEDI). Summary of ICD-10 Summit: developing an industry action plan for ICD-10 implementation. 2014 https://web.archive.org/web/20160817182515/https://www.wedi.org/workgroups/icd-10/resources/2014/05/06/summary-of-icd-10-summit-developing-an-industry-action-plan-for-icd-10-implementation Accessed October 16, 2019.

[19] Boulton C. Medical code delay costing hospitals big bucks – WSJ Blog. *Dow Jones Institutional News* 2014 May 2. https://blogs.wsj.com/cio/2014/05/02/medical-code-delay-costing-hospitals-big-bucks/ Accessed October 16, 2019.

[20] Discharge-to-bill time improves as ICD-10 transition progresses. Health Care Collector: The Monthly Newsletter for Health Care Collectors 2016; 29 (12): 3–5.

[21] Rudman WJ, Jackson K, Shank P, et al Perceived effects of ICD-10 coding productivity and accuracy among coding professionals. Perspect Health Inf Manag 2016: 1-10. https://perspectives.ahima.org/perceived-effects-of-icd-10-coding-productivity-and-accuracy-among-coding-professionals/. Accessed October 16, 2019.

[22] Stitcher S, Lawrence H. From ICD-9 to ICD10: A Comparative Analysis of Coding Audit Findings in Year One. Cambridge, MD: Maryland HFMA Fall Institute; 2016 https://hfmamd.starchapter.com/downloads/46TH_ANNUAL_INSTITUTE/3__horizon_presentation__hfma_2016_sep22.pptx Accessed October 16, 2019.

[23] Boyd AD, Li JJ, Burton MD, et al The discriminatory cost of ICD-10-CM transition between clinical specialties: metrics, case study, and mitigating tools. J Am Med Inform Assoc 2013; 20 (4): 708–17. 23645552 10.1136/amiajnl-2012-001358 PMC3721160

[24] Boyd AD, Li J, Kenost C, et al ICD-10 procedure codes produce transition challenges. AMIA Jt Summits Transl Sci Proc 2018; 2017: 35–44. PMC5961828 29888037

[25] Gibson T, Casto A, Young J, et al Impact of ICD-10-CM/PCS on research using administrative databases. HCUP Methods Series Report # 2016-02 ONLINE. July 25, 2016. U.S. Agency for Healthcare Research and Quality. http://www.hcup-us.ahrq.gov/reports/methods/methods.jsp Accessed October 16, 2019.

[26] Mainor AJ, Morden NE, Smith J, et al ICD-10 coding will challenge researchers: caution and collaboration may reduce measurement error and improve comparability over time. Med Care 2019; 57 (7): e42–e46. 30489544 10.1097/MLR.0000000000001010 PMC6535377

[27] Yoon J, Chow A. Comparing chronic condition rates using ICD-9 and ICD-10 in VA patients FY2014-2016. BMC Health Serv Res 2017; 17 (1): 572. 28818082 10.1186/s12913-017-2504-9 PMC5561575

[28] Kelley AS, Ferreira KB, Bollens-Lund E, et al Identifying older adults with serious illness: transitioning from ICD-9 to ICD-10. J Pain Symptom Manage 2019; 57 (6): 1137–42. 30876955 10.1016/j.jpainsymman.2019.03.006 PMC11200201

[29] García-Palmieri MR. Status of cardiovascular disease in Puerto Rico. P R Health Sci J 2004; 23 (1): 35–8. 15125217

[30] Hoyert DL. Maternal mortality and related concepts. Vital Health Stat 3 2007; 33: 1–13. 17460868

[31] Slabodkin G. ICD-10 transition successful so far, but patient care takes hit. *Health Data Management (Online)* 2015 Dec 1. https://www.healthdatamanagement.com/news/icd-10-transition-successful-so-far-but-patient-care-takes-hit Accessed October 16, 2019.

[32] American Medical Association (AMA). Frightening cost estimates for ICD-10 should make regulators listen. 2014 https://www.ama-assn.org/practice-management/claims-processing/frightening-cost-estimates-icd-10-should-make-regulators Accessed October 16, 2019.

[33] American Association of Family Physicians (AAFP). AAFP joins dozens of physician groups urging CMS to halt ICD-10 implementation. 2013 https://www.aafp.org/news/practice-professional-issues/20130104stopicd10.html Accessed October 16, 2019.

[34] Nachimson Advisors. The cost of implementing ICD-10 for physician practices—updating the 2008 Nachimson Advisors Study: a report to the AMA. 2014 http://docs.house.gov/meetings/IF/IF14/20150211/102940/HHRG-114-IF14-Wstate-TerryW-20150211-SD001.pdf Accessed October 16, 2019.

