# Supplementary material for: A narrative review of the impact of the transition to ICD-10 and ICD-10-CM/PCS
Source: JAMIA Open. 2019 Dec 26;3(1):126–31. doi: 10.1093/jamiaopen/ooz066 (PMC7309233; doi:10.1093/jamiaopen/ooz066)
Supplement: ooz066_Supplementary_Data [file ooz066_supplementary_data.zip › Supplementary File 1.docx]

**SUPPLEMENTARY FILE 1: Search Strategies**

**PubMed**

((("International Classification of Diseases"[mh] OR "International Classification of Diseases"[tiab]) AND ("10"[tiab] OR ten[tiab] OR tenth[tiab] OR 10th[tiab] OR "version 10"[tiab] OR "tenth revision"[tiab] OR "10th revision"[tiab])) OR ICD10[tiab] OR ICD-10[tiab] OR ICD10CM[tiab] OR ICD10-CM[tiab] OR ICD-10-CM[tiab] OR ICD10PCS[tiab] OR ICD10-PCS[tiab] OR ICD-10-PCS[tiab]) AND (change[tiab] OR conversion[tiab] OR convert[tiab] OR converted[tiab] OR converting[tiab] OR crosswalk[tiab] OR crosswalks[tiab] OR implementation[tiab] OR implemented[tiab] OR implementing[tiab] OR map[tiab] OR mapped[tiab] OR mapping[tiab] OR maps[tiab] OR migration[tiab] OR migrations[tiab] OR rollout[tiab] OR switch[tiab] OR switched[tiab] OR switching[tiab] OR transition[tiab] OR transitioned[tiab] OR transitioning[tiab] OR translating[tiab] OR translation[tiab] OR translations[tiab]) AND ("Change Management"[mh] OR "Cost Control"[mh] OR "Cost-Benefit Analysis"[mh] OR "Costs and Cost Analysis"[mh] OR "Data Accuracy"[mh] OR "delivery of health care"[mh] OR "Diffusion of Innovation"[mh] OR "disease management"[tiab] OR "fraudulent claims"[tiab] OR "Health Care Costs"[mh] OR "Health Expenditures"[mh] OR "Health Information Interoperability"[mh] OR "insurance claim reporting"[mh] OR "insurance claim review"[mh] OR "Insurance, Health, Reimbursement"[mh] OR "lessons learned"[tiab] OR "morbidity/statistics and numerical data"[mh] OR "morbidity/trends"[mh] OR "mortality/statistics and numerical data"[mh] OR "mortality/trends"[mh] OR "Organizational Innovation"[mh] OR "Personnel Management"[mh] OR "Population Surveillance"[mh] OR "Quality Indicators, Health Care"[mh] OR "quality of health care"[mh] OR "rejected claims"[tiab] OR "system change"[tiab] OR "system changes"[tiab] OR "Time Factors"[mh] OR "Workforce"[mh] OR accuracy[tiab] OR Administrative data[tiab] OR advantage[tiab] OR advantages[tiab] OR align[tiab] OR alignment[tiab] OR beneficial[tiab] OR benefit[tiab] OR benefits[tiab] OR burnout[tiab] OR “Burnout, Psychological”[mh] OR challenge[tiab] OR challenges[tiab] OR comparability[tiab] OR concordance[tiab] OR cons[tiab] OR consequence[tiab] OR consequences[tiab] OR cost[tiab] OR costs[tiab] OR discontinuities[tiab] OR discontinuity[tiab] OR "economics"[mh] OR “economics”[sh] OR "efficiency"[mH] OR error[tiab] OR errors[tiab] OR financial[tiab] OR harms[tiab] OR impact[tiab] OR implication[tiab] OR Implications[tiab] OR interoperability[tiab] OR issues[tiab] OR limitation[tiab] OR limitations[tiab] OR payment[tiab] OR payments[tiab] OR personnel[tiab] OR problem[tiab] OR problems[tiab] OR productivity[tiab] OR pros[tiab] OR reimbursement[tiab] OR time[tiab] OR training[tiab] OR validation[tiab] OR workforce[tiab] OR “case mix”[tiab] OR “case mixes”[tiab] OR casemix[tiab] OR casemixes[tiab] OR Diagnosis-Related Groups[mh] OR DRG[tiab] OR DRGs[tiab] OR “diagnosis related groups”[tiab] OR “diagnosis-related groups”[tiab] OR “diagnosis related group”[tiab] OR “diagnosis-related group”[tiab] OR “Patient Generated Health Data”[mh]) AND English[la]

*Abbreviations:* [mh] = medical subject headings; [tiab] = title/abstract keywords; [la] = language

**Web of Science:**

TI=(((("International Classification of Diseases" OR ICD) NEAR (10 OR ten OR tenth OR 10th OR “version 10” OR “10th revision” OR “tenth revision”)) OR ICD10 OR ICD-10 OR ICD10CM OR ICD10-CM OR ICD-10-CM OR ICD10PCS OR ICD10-PCS OR ICD-10-PCS)) AND TS=((change OR conversion OR convert OR converted OR converting OR crosswalk OR crosswalks OR implementation OR implemented OR implementing OR map OR mapped OR maps OR mapping OR migration OR migrations OR rollout OR switch OR switched OR switching OR transition OR transitioned OR transitioning OR translating OR translation OR translations) AND ("change management" OR cost OR costs OR accuracy OR delivery OR innovation OR diffusion OR "disease management" OR fraudulent OR expenditures OR interoperability OR “claim reporting” OR “claim review” OR reimbursement OR "lessons learned" OR morbidity OR mortality OR surveillance OR quality OR rejected OR rejection OR rejections OR "system change" OR "system changes" OR time OR workforce OR “administrative data” OR advantage OR advantages OR align OR alignment OR beneficial OR benefit OR benefits OR burnout OR challenge OR challenges OR comparability OR concordance OR cons OR consequence OR consequences OR discontinuities OR discontinuity OR economics OR efficiency OR error OR errors OR financial OR harms OR impact OR implication OR implications OR issues OR limitation OR limitations OR payment OR payments OR personnel OR problem OR problems OR productivity OR pros OR training OR validation OR “case mix” OR “case mixes” OR casemix OR casemixes OR “diagnosis-related groups” OR “diagnosis-related group” OR DRG OR DRGs OR “diagnosis related group” OR “diagnosis related groups”))

Refined by: LANGUAGES: (ENGLISH)

*Abbreviations:* TI = Title; TS = Topic search (includes title, abstract, keywords); NEAR = within 15 characters.

**Business Source Complete:**

TI ((("International Classification of Diseases" OR ICD) AND (10 OR ten OR tenth OR 10th OR “version 10” OR “10th revision” OR “tenth revision”)) OR ICD10 OR ICD-10 OR ICD10CM OR ICD10-CM OR ICD-10-CM OR ICD10PCS OR ICD10-PCS OR ICD-10-PCS) AND TI (change OR conversion OR convert OR converted OR converting OR crosswalk OR crosswalks OR implementation OR implemented OR implementing OR map OR mapped OR maps OR mapping OR migration OR migrations OR rollout OR switch OR switched OR switching OR transition OR transitioned OR transitioning OR translating OR translation OR translations)

*Abbreviation:* TI = Title
