# Supplementary material for: A narrative review of the impact of the transition to ICD-10 and ICD-10-CM/PCS
Source: JAMIA Open. 2019 Dec 26;3(1):126–31. doi: 10.1093/jamiaopen/ooz066 (PMC7309233; doi:10.1093/jamiaopen/ooz066)
Supplement: ooz066_Supplementary_Data [file ooz066_supplementary_data.zip › Supplementary File 2_2019-10-21_clean.docx]

**SUPPLEMENTARY FILE 2: Additional Studies Addressing the Impact**

**Contents**

[Productivity 2](#_Toc22217720)

[Costs 3](#_Toc22217721)

[Training Costs 3](#_Toc22217722)

[Conversion Costs 4](#_Toc22217723)

[Reimbursement 4](#_Toc22217724)

[Claims Rejection 4](#_Toc22217725)

[Revenue Impact 6](#_Toc22217726)

[Billing Time 6](#_Toc22217727)

[Coding Accuracy 7](#_Toc22217728)

[Mapping 9](#_Toc22217729)

[Morbidity Surveillance 10](#_Toc22217730)

[Mortality Surveillance 13](#_Toc22217731)

[References 15](#_Toc22217732)

## Productivity

**United States**

Citing data from Himagine Solutions, Landi described in a November 2015 article a loss in productivity for large hospitals on both inpatient and outpatient sides since the October 2015 ICD-10 implementation.[1] Specifically, large hospitals reported productivity losses of 30 to 45 percent in the inpatient setting and 20 to 40 percent in the outpatient setting following ICD-10-CM/PCS implementation.[1]

Landi also reported on a follow-up to a small HIM Benchmark Report survey of healthcare providers. The original survey took place in October 2015, but results were not provided, nor were details on the timing of the follow-up. The follow-up survey found an average 40% inpatient productivity decrease and a 10 to 35% outpatient productivity decrease for large academic facilities.[1] For community hospitals with fewer than 250 beds, inpatient productivity reductions ranged from 22 to 33 percent and outpatient productivity reductions ranged from 35 to 40 percent.[1]

A Navicure survey of 360 physician practices in December 2015 found that nearly half of respondents (48%) reported an initial decrease in staff productivity as their top issue after the ICD-10 transition.[2] Fifteen percent of clinical staff and 13% of administrative staff reported a significant impact on productivity, and 13% of administrative and clinical staff reported that their productivity rates had still not recovered two months after implementation.[2]

A March 2016 Workgroup for Electronic Data Interchange (WEDI) member survey that included 66 respondents (13 working at vendors or clearinghouses, 23 working at health plans, and 30 working at provider organizations) reported that ICD-10 had a mostly neutral impact on productivity for vendors and health plans.[3] The majority of providers reported a slight decrease in productivity, especially in coding and clinical documentation.[3]

Rudman et al. reported coders’ perceptions of productivity and accuracy following ICD-10 implementation based on phone interviews that were conducted with 156 AHIMA members identifying as coding personnel and holding coding certifications.[4] Overall, around 74% (n = 115) of respondents reported experiencing productivity changes, with 106 respondents reporting a perceived productivity decrease of 24% on average and 9 reporting a perceived productivity increase of 31% on average. The results showed overall that, while productivity was reported to have decreased 14.15%, accuracy was only reported to have decreased by 0.65%, based on 156 individuals’ reports of perceived changes. The researchers concluded that “the implementation of ICD-10 has led to a perceived decrease in productivity but has had no effect on accuracy of coding.”[4] The date of the survey was not indicated in the report.

Prior to ICD-10-CM/PCS implementation, Weems and colleagues evaluated the potential impact of the transition on coding productivity.[5] The study took place over seven months in 2012 at several Veterans Health Administration sites and included as participants ICD-9-CM coders who had received ICD-10-CM/PCS training. Results for inpatient and outpatient settings were analyzed separately. A 64.5% decrease in productivity for inpatient coding and 6.7% decrease in productivity for ambulatory care coding was observed. The ambulatory care coding study included 1,024 records and was based on data from 10 coders at 11 participating sites. The study reported an average of 6.36 minutes to code ambulatory care records in ICD-9-CM and 6.78 minutes to code the records in ICD-10-CM. The study of inpatient coding included 317 records from 13 coders at 7 sites. An average time of 26.90 minutes was needed to code records in ICD-9-CM, and 44.26 minutes was needed to code records in ICD-10-CM/PCS. The greater time needed for coding inpatient records was attributed to the large differences between ICD-10-PCS and ICD-9-CM.[5]

The American Hospital Association (AHA) and American Health Information Management Association (AHIMA) conducted ICD-10-CM field testing with 169 volunteer health information management professionals in June and August of 2003.[6] Participants coded an average of 37 records each (6,177) from both inpatient and outpatient encounters during the testing. Across all records, the average time to code a record in ICD-9-CM was 6.37 minutes, while the average time per record in ICD-10-CM was 12.14 minutes; however, no difference in time to code was observed for 58.6% (n = 3,616) of the records.[6]

**International**

In an article describing the implementation of the Australian modification of ICD-10 (ICD-10-AM) in Australia, Innes and colleagues (2000) reported, based on anecdotal reports, that “a period of at least 12 weeks is required for clinical coders to adjust to the new classification and approximate previous levels of productivity.”[7]

For Humber River Regional Hospital in Canada, which included 605 beds, the transition to ICD-10, Canada (ICD-10-CA) and The Canadian Classification of Health Interventions (CCI) led to a decrease in coding productivity.[8] While improvements in productivity were seen at 3 to 6 months post-implementation, productivity levels were still not back to pre-ICD-10 levels almost a year after ICD-10 implementation.[8]

Some differences to consider when comparing the Australian and Canadian experiences with the United states are that the Australian implementation occurred in the mid-1990s, prior to the widespread use of computers and electronic coding.[9] By contrast, ICD-10-CA in Canada was implemented at the same time as electronic coding systems which brought additional challenges beyond the coding transition itself.[9]

## Costs

### Training Costs

Kravis and colleagues (2014) reported on projected ICD-10 conversion costs for small physician practices (defined as 3 physicians plus 2 staff members affected by ICD-10 conversion) based on published reports and survey data.[10] Costs related to training (including the coding book, education for coders and physician training) along with end-to-end testing of claims were projected to range from $1,960 to $5,900. These estimates include program costs and cost of personnel time. The article cites specific costs for the following:

- Online clinician documentation and coding training: $50 to $300 for training in a particular specialty for three hours; a free educational website is also available
- ICD-10 Diagnoses Code book: downloadable for free; available for purchase for $70 to $300; ICD-10 iPhone App for $1.99
- Comprehensive staff training on ICD-10: $350 to $700 (per published national organization and vendor prices); cost of comprehensive training at Michigan State Medical Society is $400 for physicians and staff.[10]

### Conversion Costs

WEDI conducted a survey of members in March 2016, gathering responses from 66 individuals: 13 working at vendors or clearinghouses, 23 working at health plans, and 30 working at provider organizations.[3] The delays to implementation of ICD-10 increased cost. About half of responding vendors and health plans reported costs that were on target their expectations, while the remaining half of respondents was roughly equally divided between indicating that costs were higher or lower than expected. WEDI also reported that, while vendors and providers did not expect return on investment (ROI) from ICD-10 implementation, health plan respondents had mixed expectations, with about half expecting an ROI and half not anticipating any ROI.[3]

Buckholtz (2014), vice president for ICD-10 education and training at AAPC, reports on the average implementation costs per provider in a June 2014 ICD10monitor.com article.[11] The article notes that AAPC had trained over 90,000 people in ICD-10, and that they had asked past clients about the true costs for ICD-10 implementation in their practices or facilities. The costs were based on “how much vendors charged, how much they spent on education, and how much was spent on consultants”.[11] The article reports an average cost per provider of $1600, with average implementation costs based on practice size as follows:

- Small practices (<10 providers) - $750 per provider
- Medium practices (10-49 providers) - $575 per provider
- Large practices or organizations (50+ providers) - $3500 per provider

The article further notes that, “These are actual costs that do not account for staff time spent during training and education, and the costs represent clients who were able to provide us their data”.[11]

A white paper prepared by the HayGroup on behalf of America’s Health Insurance Plans (AHIP) projected, based on a literature review, “A reasonable preliminary estimate of the total cost to the healthcare system would be $3.2 to $8.3 billion,” with cost estimates for the Medicare program projected to be “$200 and 220 million” and state Medicaid program costs estimated at “$1 to $3 million each.”[12]

Jalilvand et al. (2018) noted that local/institutional costs of the transition include training, policy and procedure updates, and changes in software to accommodate the new complexity of ICD-10 compared to ICD-9.[13]

Cascardo’s 2014 management brief recommended that, given projected delays in reimbursement following implementation of ICD-10, “it is advisable [for practices] to accumulate at least three to six months of income in advance.”[14]

## Reimbursement

### Claims Rejection

A report from the Centers for Medicare & Medicaid Services (CMS) of Medicare Fee-for-Service claims based on data from October 1-27, 2015, found little change in claims rejected relative to historical baseline.[15] Consistent with historical data, 4.6 million claims were submitted per day. Likewise, claims rejected due to incomplete or invalid information remained constant with historical baselines, at 2.0% of total claims submitted. Claims rejected were slightly up to 10.1% from a historical baseline of 10%. Claims rejected for invalid ICD-10 codes were down to 0.09% from the historical baseline during end-to-end testing of 0.17%, and claims rejected for invalid ICD-9 codes were also down to 0.11% from 0.17%. Historical claims rejection data were based on 2015 end-to-end testing, as this information was not previously collected by CMS. One important limitation the report notes is that “Medicaid claims can take up to 30 days to be submitted and processed by states”; the report indicates more information about the transition would be available in November.[15]

A February 2016 CMS update reported metrics for quarter 4 of calendar year (CY) 2015; as with the October report, ICD-9 and ICD-10 claims rejection data were estimated from 2015 end-to-end testing but all other metrics were based on historical claims data.[16] Again, total claims submission was reported at 4.6 million per day, consistent with baseline, and 1.9% of claims were rejected in Q4 CY 2015 compared with the historical baseline of 2% of total claims submitted. In Q4 CY 2015, 0.07% of total ICD-10 and ICD-9 claims were rejected, compared with an estimated historical baseline of 0.17% based on 2015 CMS end-to-end testing. Of total claims processed, 9.9% were denied in Q4 CY 2015 compared with the historical baseline of 10% of total claims processed.[16]

AppRev, a company providing revenue cycle support for healthcare providers, examined denial rates and other financial data from their customers in the last three months of ICD-9 and the first three months of ICD-10.[17] While the analysis showed “little change” in authorization denials, there a consistent increase in medical necessity denials was found, and some hospitals reported that these denials doubled. Medical necessity denials were attributed to errors in Medicare National Coverage Determinations and Local Coverage Determinations.

The Cooperative Exchange ICD-10 Work Group, whose member companies represented more than 80% of the clearinghouse industry, reported metrics of claims, rejections, and denials based on data from October and November 2015.[18] Sixty-two percent of 203,364,944 analyzed claims from October 2015 were in ICD-10, while 88% of 220,254,548 claims in November 2015 were in ICD-10. ICD codes were rejected by payers at the 999/277 level at a rate of 1.5% in October and 1.1% in November. The overall ICD denial rate trended down from 2.8% in October to 2.2% in November.[18]

In a Healthcare Billing and Management Association (HBMA) survey of 38 member billing companies, 12 companies indicated they had no change in pending claims adjudication, 17 reported an increase of 10% or less, and 7 reported a greater than 10% increase.[19] For days from claim submission to adjudication, 14 companies reported no change and 13 companies reported delays of up to 14 days. Additionally, “Nine companies reported issues with various payors denying unspecified diagnosis codes. Eighteen companies reported an increase of up to 10 percent in coding error denials. A dozen companies reported no increase, and two companies reported a decrease in coding error denials.”[19] Further, denials increased due to provider coding errors for 10 companies, but 19 companies reported no increase in denials for provider coding errors. Nine companies reported provider documentation deficiencies resulting in denials, while 17 companies reported no increase in denials due to provider documentation issues.[19]

The Crowe firm evaluated the impact of the ICD-10 conversion on key performance indicators related to billing and coding, accounts receivable (AR), and denials based on patient accounting systems (PAS) data from Crowe Revenue Cycle Analytics benchmarking participants.[20] A small decrease in average initial denial rate was observed at the time of ICD-10 conversion. The report provides denials impact data from September 2014 through March 2016.[20]

A white paper from HealthAPT (2017), the CMS Chronic Condition Warehouse (CCW) contractor, evaluated the impact of the ICD-9-CM to ICD-10-CM transition on the volume of Medicare fee-for-service claims received by CCW by comparing rates from the last four months of 2014 with the last four months of 2015.[21] The analysis showed that CCW “experienced an initial decrease in the number of Medicare fee-for-service claims across all claim types compared to the prior year (October 2014),” generally “in the 1-2% range, indicating that there was minimal impact on receiving claims in the CCW.”[21] By November/December 2015, claims volumes had increased to be “higher than the previous year for all claim types except inpatient, skilled nursing facility, and hospice.” Changes in claims volumes for September-December 2014 compared with the same period in 2015 are shown in Table 1 in the white paper.[21]

A March 2015 *Medical Economics* article reported data from an American Academy of Professional Coders (AAPC) poll of its members regarding the success of November 2014 ICD-10 testing by the Centers for Medicare and Medicaid Services (CMS).[22] The article reported that the AAPC poll found, "72% had no claims denied during the test period, and 16% experienced a rejection rate of less than 10%.”[22]

### Revenue Impact

The December 2015 Navicure survey found, for 60% of 360 respondents from physician practices, monthly revenue was not impacted after October 1, 2015; however, a revenue decrease of up to 20% was seen by 34% of respondents, 5% of respondents experienced a revenue decrease of 21-40%, and 1% experienced a revenue decrease of 41-60%.[2]

Mills and colleagues (2011) estimated the impact of the ICD-10 transition on Medicare payments for inpatient services.[23] Using FY 2009 Medicare Provider Analysis and Review (MedPAR) data, the authors mapped native ICD-9-CM MS-DRGs to native ICD-10-CM MS-DRGs and estimated the payment impact. Although 1.68% of patients were reassigned to a different MS-DRG in the mapping, “payment increases and decreases due to the changes in MS-DRG assignment essentially netted out, resulting in a minimal impact on aggregate payments to hospitals (+0.05 percent) and on the distribution of payments across hospital types (-0.01 to +0.18 percent).”[23]

An updated version of this report utilizing MS-DRG version 32 and FY 2013 MedPAR data was conducted by CMS in 2015. Slightly fewer patients were reassigned to a different MS-DRG in the mapping, 1.07%.[24] Again, the net change in payment to hospitals was minimal: “The net payment change due to differences in MS-DRG assignment was -0.04 of a percent (i.e., 4 one-hundredths of one percent of the ICD-9 based MS-DRG payments)” and therefore, “estimated payment increases and decreases due to changes in MS-DRG assignment essentially netted out.”[24]

### Billing Time

The *Hospital Accounts Receivable Analysis (HARA) Report* found a decrease in average days from discharge to billing time, from 12.23 days in the third quarter to 9.71 days in the fourth quarter of 2015.[25–27] However, average days from discharge-to-bill increased in the first quarter of 2016 to 11.96 days.[27]

The Crowe analysis also found an increase in average inpatient discharge and not final billed (DNFB) days of 10.1 percent in October to December 2015 compared with the same period in 2014.[20] In the period from February to March 2016, average inpatient DNFB days were also 6.2 percent higher compared with the same period in 2015.[20]

Jalilvand et al. (2018) estimated revenue impact for a multi-hospital radiology practice by comparing average monthly days in accounts receivable per month for the year before and following the October 2015 implementation.[13] The authors reported that “no measurable delay in revenue collections in the 12 months after the transition” occurred (“accounts receivable showed no significant difference in the 12 months before and after ICD-10 implementation (P = .07)”).[13] Average number of days in accounts receivable was 36.4 days (range: 33 to 40) in the 12 months prior to implementation, compared with 34.9 days (range: 33 to 36) in the 12 months after.[13]

## Coding Accuracy

**United States**

Results from Central Learning’s 3^rd^ Annual Coding Contest in July-August 2018 showed an overall inpatient coding accuracy of 57.5%, and outpatient coding accuracy of 42.5%.[28] An undisclosed number of coders from 47 states coded 4471 “real medical record cases.” The coders reported holding an AHIMA certification (61%), an American Academy of Professional Coders (AAPC) certification (26%), or did not specify certification (3%). The 2018 results are reported in the context of 2016 and 2017 coding contest data. Overall inpatient coding accuracy was 55% in 2016, 61% in 2017, and 57.5% in 2018. The decline from 2017 to 2018 was attributed to a 42% increase in participation from inpatient coders with less than 5 years of experience. Overall outpatient coding accuracy was 38% in 2016, 41% in 2017, and 42.5% in 2018.[28]

For the previous year’s contest, in which contest coders (99% of whom identified as AHIMA or AAPC certified coders) coded 1,636 cases, accuracy for diagnosis-related groups (DRG) coding was also reported at 71% when using the same 5 cases as the previous year (compared with 72% accuracy in 2016) and 73% for 5 new cases.[29] Areas with high potential revenue loss based on the results included “DRG 455 combined anterior/posterior spinal fusion w/o cc/mcc,” with a “potential revenue loss at -$4,248 per case,” and “DRG 871 septicemia or severe sepsis w/o mv >97 hrs. w/ mcc ($3,794 per case).”[29] The contest results also showed, “Higher productivity decreased inpatient coding accuracy by -25.4 percent” and “outpatient coding accuracy by -20.3 percent.”[29]

Central Learning performed an assessment of ICD-10 coding accuracy in September 2016.[30] More than 550 coders provided codes for 1859 “real medical record cases,” including inpatient, ambulatory surgery, and emergency department cases.[30] Coding accuracy was automatically scored using an “answer key based on published coding guidelines.”[30] No details on credentials or experience of the participating coders were provided. Average coding accuracy on inpatient cases was 55%, ambulatory surgery 46%, and emergency department 33%. Coders who were more productive than ICD-9 productivity benchmarks were found to be less accurate, and external cause codes were not consistently assigned to emergency department cases.[30]

Tkacik (2016) reports “coder performance data as measured across 50 health systems and 300 coders” in the Central Learning system as of June 30, 2016.[31] The Central Learning system “electronically assesses coder knowledge using real medical record cases and expert-verified answer keys.”[31] Coding accuracy in Q1 (January-March 31, 2016) was compared with Q2 (April 1-June 30) and showed slight increases; inpatient coding accuracy improved from 83.1% in Q1 to 84.2% in Q2, ambulatory coding improved from 80.8% to 83%, and emergency services coding improved from 85.6% to 88.3%. Five areas were identified for which coding accuracy was “below acceptable levels” (with accuracy between 50% and 65% in Q1); however, all five areas showed improvement in Q2 over Q1, with the lowest accuracy category at 65.3% in Q2. See the “High Risk Coding Accuracy Categories” table in the article for more detail. Please note that this study reports outcomes based on results of coder knowledge assessments, not actual claims data.[31]

In the 2003 American Hospital Association (AHA) and American Health Information Management Association (AHIMA) ICD-10-CM field testing, a subset of 360 records (5.8% of 6,177 total coded records) was analyzed for coding accuracy by comparing coding from volunteer health information management professionals with validator-assigned codes.[6] The validation process revealed that 79.2% (n = 1,022) of ICD-10-CM codes assigned by participant and validator to the 360 records were in agreement, while 269 codes did not match. After further analysis, 97 disagreements were deemed to be participant coding errors and 100 to be validator coding errors; other reasons for discrepancies included missing digit(s) in assigned codes by participants (n = 25) and validators (n = 13), as well as “conflicting coding system instructions” (n = 14).[6]

**International**

In a retrospective review that included 4,008 patient charts from four Canadian teaching hospitals, Quan and colleagues (2008) assessed the validity of ICD-10 coding in comparison with ICD-9-CM.[32] The study found, “Of the 32 conditions assessed, ICD-10 data had significantly higher sensitivity for one condition and lower sensitivity for seven conditions relative to ICD-9-CM data,” with “similar sensitivity values for the remaining 24 conditions.”[32] However, the authors note the need to assess the longer-term impact, given that the analysis was performed soon after the Canadian ICD-10 implementation.[32]

Henderson and colleagues (2006) reported on the accuracy of diagnosis and procedure coding with the implementation of the ICD-10 Australian Modification (ICD-10-AM) using a random public hospital sample from Victoria, Australia, immediately following implementation in 1998-1999 (n = 7004) and after two years of implementation using a second random sample from 2000-2001 (n = 7631) to assess stabilization effects.[33] Accuracy was assessed using auditor coding as the reference standard. The number of codes used was similar among the comparison groups; “12% to 13% of records differed by more than 1 code” for diagnoses and “only 1% to 2% of records differed” for procedures.[33] The investigators also reported, “Agreement of the principal diagnosis code was 85% at the 3-digit level and 79% at the 4-digit level in 1998-1999” and “improved to 87% and 81% in 2000-2001,” while agreement for principal procedure code was 85% at the 5-digit level and 81% at the 7-digit level in 1998-1999 and 83% and 80%, respectively, in 2000-2001.[33] In a review of studies reporting audits of ICD-9 accuracy, the authors found lower rates of agreement for ICD-9; for instance, “ICD-9-CM audit data from 1993-1994 in Victoria, Australia, indicated that the agreement rate between principal diagnosis at the 3-digit level was 73%.”[33] Based on these findings, the authors conclude, “The transition to ICD-10 has occurred with no loss of data quality, with data showing a high level of reliability and adherence to coding standards.”[33]

Two studies [34,35] have also reported on the accuracy of ICD-10 coding for specific conditions (stroke and pulmonary embolism) in single centers in Canada. Burles et al. (2017) found, based on data from patient visits to the Calgary Emergency Department, that 17.7% of pulmonary embolism ICD-10 codes (257/1453) were “incorrectly assigned” based on chart review but noted that ICD-10 coding had similar performance characteristics to those reported for ICD-9 in prior studies.[35] Kokotailo et al. (2005) reported, based on a random selection of charts with hospital discharge data from three Calgary adult care hospitals (461 charts from the ICD-9 coding period of 2000/2001 and 256 charts from the ICD-10 coding period of 2002/2003), “ICD-9 coding was excellent with 90% (CI_95_, 86 to 92) correct; κ=0.86 (CI_95_, 0.81 to 0.91),” and “ICD-10 was similarly good with 92% (CI_95_, 88 to 95) of strokes correctly coded; κ=0.89 (CI_95_, 0.82 to 0.96),” but “ICD-10 was not better than ICD-9; *P*=0.865.”[34]

## Mapping

In a retrospective analysis of patient data from a single institution over a 6-month period in 2016, Angiolillo and colleagues (2019) assessed the impact of the ICD-9 to ICD-10 transition on eleven Choosing Wisely metrics.[36] The patient data was in ICD-10; however, the rules for Choosing Wisely metrics were defined using ICD-9. The study compared the performance of three different algorithms and found that the prevalence of avoiding Choosing Wisely services was similar across groups. Estimates of waste due to the eleven services varied based on the method from $871,049 to $951,829. The study found that General Equivalence Mappings (GEMs) could be used to apply ICD-9 based metrics to ICD-10 data. However, they found that “for the five metrics which identified denominator cases by diagnosis codes alone, mapping can introduce substantial error.”[36]

Noting that the delay in US adoption of ICD-10 was largely due to “anticipated adverse impacts on revenue and cash flow,” Jalilvand et al. (2018) undertook a retrospective observational study of 673,600 radiology services performed during 12 months between 2015-2016 by 179 radiologists in multiple radiology departments within a large healthcare system which had taken proactive steps for the 2015 transition.[13] The authors believe that the projections and preparation from the previous transition would be applicable and useful for the ICD-11 transition in terms of *code conversion impact factor* (CCIF) and impact on revenue. ICD-10 CCIFs were calculated as (number of actual 90th percentile ICD-10 codes used during the post implementation period of December 2015 to September 2016 / number of 90th percentile ICD-9 codes used in the 2014 calendar year). Analysis of the 2015 transition showed that “actual increases in the number of codes used were only a fraction of what was predicted” (predicted CCIF=5.9 and actual CCIF= 1.6).[13] For individual subspecialties, the magnitude of CCIF overestimation ranged from 157% (Breast; 1.1 predicted vs. 0.7 actual) to 823% (Musculoskeletal; 28.8 predicted vs. 3.5 actual).[13]

Della Mea et al. (2015) developed and validated a transcoding system named TranIT for mapping Italian morbidity data across ICD-9 and ICD-10.[37] At the time of writing, Italy used ICD-9-CM for morbidity data, and TranIT was used to assist in the creation of Italian ICD-10 DRGs. They used it to transcode ICD 9-CM codes in the administrative Hospital Discharge Form database to ICD-10 and evaluated the impact of the mapping on morbidity statistics in 3 regions in Italy from 2011 to 2012. A total of 3,374,323 subsets of the Hospital Discharge Form (SDOs) were analyzed, and the results showed “a large number of SDOs ICD 9-CM codes (86.36%) was transcoded automatically,” while “the remaining 12.86% of SDO ICD9-CM codes needed manual intervention” and 0.006% of SDOs could not be transcoded.[37] The researchers concluded the transition from ICD-9 to ICD-10 “could be less difficult than supposed;” however, training was needed for coders to better understand the differences between the two versions.[37]

Steindel (2010) reported that “government-developed general equivalence mappings are provided for ICD-9-CM to/from ICD-10-CM and ICD-9 volume 3 to/from ICD-10-PCS, but are not particularly helpful in that more than 95% of codes are noted as having only an approximate match.”[38] Percentage general equivalence mappings from ICD-9-CM to ICD-10-CM with an exact, approximate, or no map were 10.4%, 89.6%, and 1.2%, respectively. See table 5 of Steindel (2010) for more information about percentage general equivalence mappings between ICD-9-CM/ICD-9 volume 3 and ICD-10-CM/ICD-10-PCS.[38]

## Morbidity Surveillance

**United States**

Lind and colleagues (2019) assessed the impact of the ICD-9-CM to ICD-10-CM transition on estimates of neonatal abstinence syndrome (NAS) in a pilot project in New Mexico, Illinois, and Vermont.[39] The study found that in two of the states, the transition from ICD-9-CM code 779.5 (drug withdrawal syndrome in a newborn) to ICD-10-CM code 96.1 (drug withdrawal, infant of dependent mother) did not impact the positive predictive value for confirmed NAS cases.[39]

Stewart and colleagues conducted a series of studies to evaluate the impact of the ICD-10-CM implementation on coding of suicide attempts, self-harm, and mental health diagnoses.[40,41] Both studies used data from the Health Care Systems Research Network Virtual Data Warehouse (HCSRN VDW) from systems in the Mental Health Research Network (MHRN). Based on an analysis of data from 10 systems from April 2014 through March 2016, the investigators reported, “Diagnoses of self-inflicted injury or poisoning appeared to increase abruptly with the coding transition, and this pattern was consistent across health systems,” while diagnoses for “injury or poisoning of undetermined intent appeared to decrease with the coding transition, but this pattern varied considerably across health care systems.”[40] The authors stated that the changes in suicidal behavior coding “almost certainly represent artifacts of coding changes rather than true changes in suicidal behavior.”[40]

In a second study analyzing data from 10 sites from October 2014 through September 2016, the investigators found “the rate of psychotic disorder diagnoses other than schizophrenia decreased by almost 40 percent at the time of the transition to ICD-10-CM and remained relatively stable thereafter,” with “proportional decreases rang[ing] from approximately 30 percent at some sites to approximately 60 percent at others.”[41] However, the authors reported, “Rates of bipolar disorder, attention deficit disorder, schizophrenia and personality disorder remained stable across the transition to ICD-10-CM,” and a “small gradual increase over the entire two-year period” was observed for anxiety and eating disorder rates, while “depression rates decrease[d] in a similarly gradual way.”[41] The authors concluded that, while the ICD-10-CM transition “appears to have had a minimal impact” overall for mental health conditions, the change in psychosis diagnoses “is likely due to changes associated with EHR implementation of ICD-10-CM coding rather than an actual change in disease prevalence.”[41]

Panozzo and colleagues (2018) analyzed the impact of the implementation of ICD-10-CM coding on five health outcomes within the U.S. Food and Drug Administration’s Sentinel system for medical product safety reporting: acute myocardial infarction (AMI), angioedema, ischemic stroke, diabetes, and hypertension.[42] Methods used for identifying conditions of interest included algorithms identified in the literature or used in previous studies, and Simple Forward Mapping (SFM), Simple Backward Mapping (SBM) and Forward-Backward Mapping (FBM) GEMs. The study used data from 13 partners with a “diverse group of national and regional insurers and integrated delivery systems.”[42] The trend analysis included data from October 1, 2010 through December 31, 2016 from 172 million members representing 443 million member-years, while the coding-era analysis used data from April 1, 2016 through December 31, 2016, as well as data from the same period in 2014, from 71 million members representing 46 million member-years. The authors reported “[w]hile the incidence or prevalence of AMI and hypertension performed similarly across the ICD‐9‐CM and ICD‐10‐CM eras, the other 3 health outcomes, including angioedema, ischemic stroke, and diabetes did not demonstrate consistent results for some or all the ICD‐10‐CM definitions assessed.”[42] SBM and FBM GEMs had better performance than the SFM GEM, and in post-hoc analysis several data coding errors were identified for some of the health outcomes with inconsistent results across the transition. Overall, the authors found “the early impact of ICD‐10‐CM transition on incidence and prevalence estimated in US electronic health care databases may vary by health outcome”; thus, they recommended “testing multiple ICD‐10‐CM outcome definitions as part of routine sensitivity analysis, as well as continued monitoring of health outcomes to further assess the impact of the transition as more data accumulate.”[42]

Heslin and Barrett (2018) evaluated the presence of discontinuities in alcohol-related hospital stays after the transition to ICD-10-CM using data from the Agency for Healthcare Research and Quality Healthcare Cost and Utilization Project State Inpatient Databases from six quarters before and after the ICD-10-CM transition (2014-2017) for 17 states.[43] While the overall trend in alcohol-related diagnosis stays remained stable, substantial shifts in stays were observed for certain subgroups, including alcohol-induced mental disorders (+170.7%), alcohol abuse (-14.0%), and intoxication and toxic effects (+46.6%). A contributing factor to the change in prevalence of stays associated with alcohol-induced mental disorders was the F10.99 ICD-10-CM code for alcohol use, unspecified, with unspecified alcohol-induced disorder. A 90% drop in use of the code F10.99 was observed when the CMS grace period for using unspecified codes ended on October 1, 2016.[43]

In an interrupted time series design study, which included Kentucky hospitalization data from 2012-2017, Slavova and colleagues (2018) assessed the impact of ICD-10-CM implementation on injury hospitalization trends.[44] The study “showed an immediate significant drop in external-cause-of-injury completeness during the transition month,” which “returned to its pre-transition levels in November 2015. There was a significant immediate change in the percentage of injury hospitalizations coded for unintentional (3.34%) and undetermined intent (- 3.39%).”[44]

Sieben et al. (2018) presented findings from an evaluation of the impact of ICD-10-CM on sexual violence trends in Minnesota.[45] In an analysis of discharge data from 2010-2016 for hospitals and emergency departments affiliated with the Minnesota Hospital Association, an increase in sexual violence cases was observed between 2014 and 2016, even when using a “conservative” definition. However, further analysis suggested the increase began prior to the October 2015 ICD-10 implementation, and the authors observed, “When considering all SV cases, number of SV cases appears to be cyclical, with an increase between 2015 and 2016.”[45]

The New York State Department of Health conducted a study on the impact of the ICD-10-CM transition for injury surveillance.[46] They analyzed the data on injury diagnosis coding from 2015 Quarter 4 through 2016 Quarters 1-3 and also compared the data of 2014 Quarter 4 with 2015 Quarter 4 on a proposed external cause matrix. The authors concluded that “ICD-10-CM data quality is good,” but there were “[s]ignificant changes in outcome data.” Additionally, “Misclassification potentially occur[ed] in MVT," and “[o]pportunities [were] identified for case definition revision.”[46]

Hobbs and colleagues (2017) studied the increase in diagnosis codes between ICD-9-CM to ICD-10-CM and found ICD-10-CM’s new anatomic and physiologic specificity generated an increase in unspecified codes in all major diagnosis categories except for three categories: Infectious & parasitic, Blood and blood-forming organs & immune disorders, and Factors influencing health status.[47] They further investigated the FY2016 Massachusetts case mix inpatient acute care hospital setting and Massachusetts All Payer Claims Data (APCD) ICD-10-CM coding for trauma patients in the Prehospital Outpatient Ground and Air Ambulance Health Care setting. The most common unspecified diagnoses in ICD-9-CM were mapped to ICD-10-CM by the GEMs mapping for the top unspecified poisoning diagnosis as assigned by hospitals and top unspecified trauma diagnosis as assigned by ground and air ambulance crews. ICD-10-CM provided more detailed information for the unspecified codes examined, such as the most common cause of unspecified poisoning in their sample “T426X2A (Poisoning by other antiepileptic and sedative-hypnotic drugs, intentional self-harm, initial encounter) ranked number one providing new information on the intentional anticonvulsants for self-harm.”[47] The authors concluded that ICD-10-CM implementation will benefit public health injury surveillance, improve “the calculation of disease and injury incident rates,” and facilitate “aligning codes with clinical facts.”[47]

Heslin and colleagues (2017) analyzed trends in opioid-related hospitalizations following the implementation of ICD-10-CM.[48] Based on ICD-9-CM data from each quarter of 2015 and ICD-10-CM data from Q4 2015 and the first 3 quarters of 2016, the investigators found that inpatient “stays involving any opioid-related diagnosis increased by 14.1% [7514 stays] during the ICD transition – which was preceded by a much lower 5.0% average quarterly increase before the transition followed by a 3.5% average increase after the transition.”[48] This increase was attributed in part to an increase in the number of “unspecified use” diagnoses, “accounting for 53.8% of the overall increase.”[48]

HealthAPT (2017) conducted an analysis of prevalence estimates for conditions included in the CMS Chronic Condition Data Warehouse (CCW).[21] Forward and backward mapping using General Equivalence Mappings (GEMs) was conducted for code conversion, with results verified using the ICD-10 codebook. For the 27 CCW chronic conditions and 33 other CCW chronic conditions and disabling conditions, “percentage change in prevalence between 2014 and 2015 was consistent with the average annual change from 2010-2014” based on 100% of Medicare fee-for-service claims.[21] Exceptions for which deviations were observed included chronic kidney disease (2014/2015 percentage change of 1.10 vs. average annual percentage change of 1.00), asthma (1.63 vs. 1.00), spinal cord injury (1.30 vs. 1.02), cystic fibrosis and other metabolic developmental disorders (1.20 vs. 1.05), and personality disorders (1.35 vs. 1.03). Upon closer analysis, some of these changes were attributed to changes in the algorithm due to mapping (e.g., three chronic obstructive pulmonary disease codes were added to the asthma algorithm); however, prevalence changes were observed for other conditions such as chronic kidney disease that did not have significant algorithm differences across coding versions, and some conditions, such as hip/pelvic facture, had large numbers of additional codes but showed little change in prevalence.[21]

Poltavskiy and Romano (2016) describe the development of an SAS macro for differentiation of ICD-9-CM and ICD-10-CM, as well as ICD-9-CM and ICD-10-PCS records, with the rationale that some entities (such as employee health clinics and workers compensation) were not required to switch to ICD-10; services spanning ICD-10 implementation in October 2015 may have split claims with ICD-9-CM and ICD-10-PCS codes depending on exact date of service; and methods are needed to identify coding errors and inappropriate dual coding to enable adjudication and rejection.[49] This method was found to accurately classify codes from an artificial dataset that contained both ICD-9 and ICD-10 data, as well as identify invalid procedure codes in the National Inpatient Sample (NIS) dataset of over eight million records.[49]

A 2016 presentation from Romano also reports on the utility of dual coded data.[50] Using dual coded data sets from the Washington State Department of Health (2,665 records from 8 hospitals from April to October 2013) and the University of California Davis Medical Center (5,167 records from 1 academic medical center from September 2014 to September 2015), comparability ratios for AHRQ inpatient quality indicators (IQIs) between ICD-9-CM and ICD-10-CM/PCS were estimated. Romano concludes that dual coding is valuable for training and monitoring of transition-related outcomes. Additionally, “Dual coded data helped AHRQ to identify and correct potential comparability problems for IQIs and PQIs”; however, “some comparability problems are intrinsic in how the code sets were designed” and, while “GEM mappings provide helpful relationships and alternatives…, measure developers/testers MUST add clinical and coding review.”[50]

Fenton and Benigni (2014) noted that the transition from ICD-9-CM to ICD-10-CM/PCS was expected to "result in longitudinal data discontinuities for disease and procedural reporting” based on similar occurrences in cause of death statistics when ICD-10 began being used for mortality reporting.[51] They stated that “comparability ratios are needed to … track and trend data longitudinally.”[51] To examine this hypothesis, 3,969 de-identified coded discharge records from July 2011 and July 2012 from the University of Wisconsin Hospital were assessed. Codes had to appear >80 times in the hospital data to be included in the comparison between ICD-9-CM and ICD-10-CM. Comparability factors were calculated as: (ICD-10-CM code number of occurrences / GEM-indicated matching ICD-9 code number of occurrences) multiplied by 100. Comparability Factors <100 indicate fewer cases were coded for a particular disease/condition in ICD-10 than in ICD-9, therefore indicating some measure of discontinuity. Ten of twelve Joint Commission Core Measures and 86/90 ICD-9 codes had comparability factors under 100 in the analysis. Study authors concluded that reliance on longitudinal data for reporting may require investigation of the impact that conversion from ICD-9 to ICD-10 has had on the data.[51]

Lau and colleagues (2018) reviewed fourth-quarter data from the National Ambulatory Medical Care Survey (NAMCS) in 2014 (*n* = 20,942 visit records) to assess the impact of the ICD-9-CM to ICD-10-CM transition on the reporting of diagnosis data by the National Center for Health Statistics (NCHS).[52] The records were dual-coded using both ICD-9-CM and ICD-10-CM. The study showed that “89% had a matching ICD-9-CM and ICD-10-CM code for the primary diagnosis, indicating that the transition from the ICD-9-CM to ICD-10-CM coding systems did not have a large effect on the way diagnosis codes were coded and reported in NAMCS. Among the remaining records that had mismatches, the mismatches were attributable to coding errors (5% of all visit records), uncodable diagnoses (3% of all visit records), and chapter change between the two coding systems (2% of all visit records).”[52]

**International**

In a time series analysis of injuries registered in the Swedish National Patient Register, Nilson and colleagues (2015) assessed the impact of the 1997 ICD-9 to ICD-10 conversion on external cause coding of injury morbidity.[53] The study showed “a large spike in the proportion of injury admissions registered without an external cause code in 1997, with continuing, although gradually diminishing, problems up to 2002.”[53] The most affected categories were those lacking direct code conversions between ICD-9 and ICD-10 primary codes.[53]

## Mortality Surveillance

**United States**

A retrospective study by Joyner-Grantham and colleagues (2010) assessed the impact of the ICD-10 transition on hypertension-related mortality rates in 16 states in the southeastern U.S. using CDC National Vital Statistics Reports data from 1994-2005.[54] The study found that the change in codes impacted diabetes, heart disease, and cerebrovascular disease mortality rates. The authors discuss the need to consider the impact of ICD code changes when interpreting mortality data spanning different ICD versions.[54]

McCoy et al.[55] and Sly[56] reported a decrease in asthma mortality rates in the United States around the time of ICD-10 implementation but noted this change was only partially attributable to the ICD-10 transition, as rates were already decreasing in 1997[56] and declined between 1998 and 1999 even after correcting for a comparability ratio of 0.89.[55]

**International**

International studies reporting on the impact of the ICD-10 implementation on mortality rates include an analysis of intentional and unintentional injury mortality in Italy and Norway;[57] a report of mortality trend discontinuities for COPD in France;[58] a series of studies investigating Alzheimer’s disease, Parkinson’s disease, dementia, circulatory and respiratory disease mortality trends in England and Wales;[59–62] and an analysis of suicide rates over multiple ICD implementations in 71 countries.[63]

##

## References

1. Landi H. Survey: ICD-10 coding has reduced productivity. November 12, 2015. <https://www.hcinnovationgroup.com/policy-value-based-care/news/13025971/survey-icd10-coding-has-reduced-productivity> Accessed October 17, 2019.

1. Navicure. Healthcare organization post ICD-10 implementation survey: key survey findings. January 2016. <https://web.archive.org/web/20180219123237/http:/info.navicure.com/rs/669-OIJ-380/images/Navicure-Post-ICD-10-Survey_Final.pdf> Accessed October 17, 2019.
2. Workgroup for Electronic Data Interchange (WEDI). WEDI ICD-10 post-implementation survey results released. 2016 May 9. <https://www.wedi.org/news/press-releases/2016/05/09/wedi-icd-10-post-implementation-survey-results-released> Accessed April 30, 2019.

4. Rudman WJ, Jackson K, Shank P, et al*.* Perceived effects of ICD-10 coding productivity and accuracy among coding professionals. *Perspect Health Inf Manag* 2016:1-10. <https://perspectives.ahima.org/perceived-effects-of-icd-10-coding-productivity-and-accuracy-among-coding-professionals/> Accessed October 17, 2019.

5. Weems S, Heller P, Fenton SH. Results from the Veterans Health Administration ICD-10-CM/PCS Coding Pilot Study. *Perspect Health Inf Manag* 2015;12:1b.

6. American Hospital Association (AHA), American Health Information Management Association (AHIMA). ICD-10-CM field testing project: report on findings: perceptions, ideas and recommendations from coding professionals across the nation. September 23, 2003. <http://library.ahima.org/doc?oid=61292#.XREsu49OmUm> Accessed October 17, 2019.

7. Innes K, Peasley K, Roberts R. Ten down under: implementing ICD-10 in Australia. *J AHIMA* 2000;71:52–6.

8. Johnson K. Implementation of ICD-10: experiences and lessons learned from a Canadian hospital. *IFHRO Congress & AHIMA Convention Proceedings* 2004 Oct 15. <http://library.ahima.org/doc?oid=60124> Accessed October 17, 2019.

9. Dimick C. ICD-10 postcards. Canadians, Australians share experiences with ICD-10 implementation. *J AHIMA* 2008;79:33–5.

10. Kravis TC, Belley S, Smith DM, et al*.* Cost of converting small physician offices to ICD-10 much lower than previously reported. *J AHIMA* 2014 Nov. <https://journal.ahima.org/wp-content/uploads/Week-3_PDFpost.FINAL-Estimating-the-Cost-of-Conversion-to-ICD-10_-Nov-12.pdf> Accessed October 17, 2019.

11. Buckholtz R. ICD-10 implementation – where do we really stand? *ICD10 Monit* 2014. <https://www.icd10monitor.com/exclusive-icd-10-implementation-where-do-we-really-stand> Accessed October 17, 2019.

12. Wildsmith TF. Examining the cost of implementing ICD-10. HayGroup on behalf of America's Health Insurance Plans; 2006 Oct 12. <http://www.ehcca.com/presentations/hithipaa414/3_04_1.pdf> Accessed October 17, 2019.

13. Jalilvand A, Fleming M, Moreno C, et al*.* Code conversion impact factor and cash flow impact of International Classification of Diseases, 10th Revision, on a large multihospital radiology practice. *J Am Coll Radiol* 2018 Jan;15(1 Pt A):69-74. <http://doi.org/10.1016/j.jacr.2017.08.013>

14. Cascardo D. Customer service with financial savvy: integrating customer service with the ICD-10 transition to keep patients coming back and optimize revenue. *J Med Pract Manag MPM* 2014;30:20–3.

15. Centers for Medicare & Medicaid Services (CMS). ICD-10 transition moves forward. October 2015. <https://www.cms.gov/newsroom/fact-sheets/icd-10-transition-moves-forward> Accessed October 17, 2019.

16. Slavitt A. Lessons learned: reflections on CMS and the successful implementation of ICD-10. CMS Blog. February 24, 2016. <https://web.archive.org/web/20170118123955/https://blog.cms.gov/2016/02/24/lessons-learned-reflections-on-cms-and-the-successful-implementation-of-icd-10/> Accessed October 17, 2019.

17. AppRev. ICD-9 to ICD-10: a look back: tracking the changes in the transition. May 2016. <http://apprev.com/icd10-study.php> Accessed October 17, 2019.

18. Gomez B, Kossow J. Clearinghouse realities of ICD-10. Cooperative Exchange; 2015 Dec 10.

19. Louie H. Latest results: HBMA’s ICD-10 Benchmark Survey: three rev cycle companies shuttered. <https://www.icd10monitor.com/latest-results-hbma-s-icd-10-benchmark-survey-three-rev-cycle-companies-shuttered> Accessed October 17, 2019.

20. Crowe Revenue Cycle Analytics. ICD-10 conversion results in limited performance impact for most hospitals. 2016. <https://www.crowe.com/insights/asset/i/icd-10-conversion-performance-impact> Accessed October 17, 2019.

21. HealthAPT, Chronic Condition Data Warehouse. CCW condition categories: impact of conversion from ICD-9-CM to ICD-10-CM. 2017. <https://www.ccwdata.org/web/guest/ccw-medicare-data-white-papers> Accessed October 17, 2019.

22. Coding group predicts a smooth transition to ICD-10. *Med Econ* 2015;92:15.

23. Mills RE, Butler RR, McCullough EC, et al*.* Impact of the transition to ICD-10 on Medicare inpatient hospital payments. *Medicare Medicaid Res Rev* 2011 Jun 6;1(2). <https://doi.org/10.5600/mmrr.001.02.a02>

24. Mills RE, Butler RR, Averill RF, *et al.* Impact of the transition to ICD-10 on Medicare inpatient hospital payments. *J AHIMA Website* 2015 Feb. <http://bok.ahima.org/doc?oid=301720> Accessed October 17, 2019).

25. Anonymous. Discharge-to-bill time improves as ICD-10 transition progresses. *Health Care Collect* 2016;29:3-5.

26. Anonymous. As ICD-10 transition progresses, billing time improves. *Receiv Rep N Y* 2016;31:6-8.

27. Anonymous. 2016 1st Quarter. *Hosp Acc Receiv Anal* 2016;30.

28. Central Learning. National ICD-10 coding contest results. Baltimore, MD: Central Learning; 2018. <https://www.centrallearning.com/contestresults> Accessed October 17, 2019.

29. AHIMA Staff. 2nd annual ICD-10 coding contest results. *J AHIMA* 2017. <https://journal.ahima.org/2017/10/06/2nd-annual-icd-10-coding-contest-results-sponsored/> Accessed October 17, 2019.

30. Central Learning. Central Learning releases 1st nationwide ICD-10 coder performance data. Baltimore, MD: Central Learning; 2016. <https://www.centrallearning.com/central-learning-releases-1st-nationwide-icd-10-coder-performance-data/> Accessed October 17, 2019.

31. Tkacik ED. One year of ICD-10: first half 2016 data shows coding trends and impacts. *HIM Brief* 2016. <https://www.centrallearning.com/one-year-of-icd-10-first-half-2016-data-shows-coding-trends-and-impacts/> Accessed October 17, 2019.

32. Quan H, Li B, Saunders LD, et al. Assessing validity of ICD-9-CM and ICD-10 administrative data in recording clinical conditions in a unique dually coded database. *Health Serv Res* 2008;43(4):1424–41. <https://doi.org/10.1111/j.1475-6773.2007.00822.x>

33. Henderson T, Shepheard J, Sundararajan V. Quality of diagnosis and procedure coding in ICD-10 administrative data. *Med Care* 2006;44(11):1011-9. <https://doi.org/10.1097/01.mlr.0000228018.48783.34>

34. Kokotailo RA, Hill MD. Coding of stroke and stroke risk factors using international classification of diseases, revisions 9 and 10. *Stroke* 2005;36(8):1776-81. <https://doi.org/10.1161/01.STR.0000174293.17959.a1>

35. Burles K, Innes G, Senior K, et al*.* Limitations of pulmonary embolism ICD-10 codes in emergency department administrative data: let the buyer beware. *BMC Med Res Methodol* 2017 Jun 8;17(1):89. <https://doi.org/10.1186/s12874-017-0361-1>

36. Angiolillo J, Rosenbloom ST, McPheeters M, et al*.* Maintaining automated measurement of Choosing Wisely adherence across the ICD 9 to 10 transition. *J Biomed Inform* 2019 Mar 7;93:103142. <https://doi.org/10.1016/j.jbi.2019.103142>

37. Della Mea V, Vuattolo O, Frattura L, et al*.* Design, development and first validation of a transcoding system from ICD-9-CM to ICD-10 in the IT.DRG Italian project. *Stud Health Technol Inform* 2015;210:135-9.

38. Steindel SJ. International classification of diseases, 10th edition, clinical modification and procedure coding system: descriptive overview of the next generation HIPAA code sets. *J Am Med Inform Assoc JAMIA* 2010;17(3):274-82. <https://doi.org/10.1136/jamia.2009.001230>

39. Lind JN, Ailes EC, Alter CC, et al*.* Leveraging existing birth defects surveillance infrastructure to build neonatal abstinence syndrome surveillance systems - Illinois, New Mexico, and Vermont, 2015-2016. *MMWR Morb Mortal Wkly Rep* 2019;68(7):177-80. <https://doi.org/10.15585/mmwr.mm6807a3>

40. Stewart C, Crawford PM, Simon GE. Changes in coding of suicide attempts or self-harm with transition from ICD-9 to ICD-10. *Psychiatr Serv* 2017;68(3):215-215. <https://doi.org/10.1176/appi.ps.201600450>

41. Stewart CC, Lu CY, Yoon TK, *et al.* Impact of ICD-10-CM transition on mental health diagnoses recording. *EGEMS Wash DC* 2019;**7**(1):14. <https://doi.org/10.5334/egems.281>

42. Panozzo CA, Woodworth TS, Welch EC, et al*.* Early impact of the ICD-10-CM transition on selected health outcomes in 13 electronic health care databases in the United States. *Pharmacoepidemiol Drug Saf* 2018;27(8):839-47. <https://doi.org/10.1002/pds.4563>

43. Heslin KC, Barrett ML. Shifts in alcohol-related diagnoses after the introduction of International Classification of Diseases, Tenth Revision, Clinical Modification coding in US hospitals: implications for epidemiologic research. *Alcohol Clin Exp Res* 2018;42(11):2205-13. <https://doi.org/10.1111/acer.13866>

44. Slavova S, Costich JF, Luu H, et al*.* Interrupted time series design to evaluate the effect of the ICD-9-CM to ICD-10-CM coding transition on injury hospitalization trends. *Inj Epidemiol* 2018;5(1):36. <https://doi.org/10.1186/s40621-018-0165-8>

45. Sieben A, Gaichas A, Roesler J, et al*.* Hospital-treated sexual violence in Minnesota: characterizing the effect of ICD-10-CM. 2018 Safe States Alliance Annual Meeting; 2018 Sep 6; Charleston. <https://www.safestates.org/page/2018AMPresentations> Accessed October 17, 2019.

46. Bauer M, Garnett M, Hines L. New York state findings on the impact of the ICD-10-CM transition for injury surveillance. 2018 Safe States Alliance Annual Meeting; 2018 Sep 5-7; Charleston. <https://cdn.ymaws.com/www.safestates.org/resource/resmgr/2018_annual_meeting/presentations/42_NYSDOH_ICD10CM.pdf> Accessed October 17, 2019.

47. Hobbs S, Medinus A, Fu Y, et al*.* Profiling by care setting information gain in the transition from ICD-9-CM to ICD-10-CM: a combined all payer claims data and administrative case mix approach. NAHDO’s 32nd Annual Conference; 2017 Oct. <https://www.nahdo.org/sites/nahdo.org/files/Sylvia%20hobbs%20-%20Final_NAHDO_HOBBS_MEDINUS_FU_HINES_with_lastRevisions.pdf> Accessed October 17, 2019.

48. Heslin KC, Owens PL, Karaca Z, et al*.* Trends in opioid-related inpatient stays shifted after the US transitioned to ICD-10-CM diagnosis coding in 2015. *Med Care* 2017;55(11):918-23.

49. Poltavskiy E, Romano P, NAHDO/UC Davis Team. 1. An updated SAS macro to differentiate ICD-9-CM and ICD-10-CM records. 2. Use of dual coded data to assess comparability ratios for ICD-based surveillance/morbidity measures. Salt Lake City: National Association of Health Data Organizations; 2016 Feb 3.

50. Romano PS. ICD-10 implementation: opportunities and challenges for health data organizations. NAHDO Annual Meeting; 2016 Oct 27.

51. Fenton SH, Benigni MS. Projected impact of the ICD-10-CM/PCS conversion on longitudinal data and the Joint Commission Core Measures. *Perspect Health Inf Manag* 2014;11:1g.

52. Lau DT, Strashny A, Phan K, et al*.* Evaluation of transition from ICD-9-CM to ICD-10-CM diagnosis coding system in the National Ambulatory Medical Care Survey. *Natl Health Stat Rep* 2018 Nov;(120):1-10.

53. Nilson F, Bonander C, Andersson R. The effect of the transition from the ninth to the tenth revision of the International Classification of Diseases on external cause registration of injury morbidity in Sweden. *Inj Prev* 2015;21(3):189-94. <https://doi.org/10.1136/injuryprev-2014-041337>

54. Joyner-Grantham J, Simmons DR, Moore MA, et al*.* The impact of changing ICD code on hypertension-related mortality in the southeastern United States from 1994-2005. *J Clin Hypertens* 2010;12(3):213-22. <https://doi.org/10.1111/j.1751-7176.2009.00232.x>

55. McCoy L, Redelings M, Sorvillo F, et al*.* A multiple cause-of-death analysis of asthma mortality in the United States, 1990-2001. *J Asthma* 2005;42(9):757-63. <https://doi.org/10.1080/02770900500308189>

56. Sly RM. Continuing decreases in asthma mortality in the United States. *Ann Allergy Asthma Immunol* 2004;92(3):313-8. <https://doi.org/10.1016/S1081-1206(10)61568-2>

57. Gjertsen F, Bruzzone S, Vollrath ME, et al*.* Comparing ICD-9 and ICD-10: the impact on intentional and unintentional injury mortality statistics in Italy and Norway. *Injury* 2013;44(1):132-8. <https://doi.org/10.1016/j.injury.2012.01.010>

58. Fuhrman C, Jougla E, Nicolau J, et al*.* Deaths from chronic obstructive pulmonary disease in France, 1979-2002: a multiple cause analysis. *Thorax* 2006;61(11):930-4. <https://doi.org/10.1136/thx.2006.061267>

59. Mylne AQ, Griffiths C, Rooney C, et al*.* Trends in Parkinson’s disease related mortality in England and Wales, 1993-2006. *Eur J Neurol* 2009;16(9):1010-6. <https://doi.org/10.1111/j.1468-1331.2009.02715.x>

60. Griffiths C, Rooney C. Trends in mortality from Alzheimer’s disease, Parkinson’s disease and dementia, England and Wales, 1979-2004. *Health Stat Q* 2006 Summer;(30):6-14.

61. Brock A, Griffiths C, Rooney C. The impact of introducing ICD-10 on analysis of respiratory mortality trends in England and Wales. *Health Stat Q* 2006 Spring;(29):9-17.

62. Griffiths C, Brock A, Rooney C. The impact of introducing ICD-10 on trends in mortality from circulatory diseases in England and Wales. *Health Stat Q* 2004 Summer;(22):14-20.

63. Pearson-Nelson BJ, Raffalovich LE, Bjarnason T. The effects of changes in the World Health Organization’s International Classification of Diseases on suicide rates in 71 countries, 1950-1999. *Suicide Life Threat Behav* 2004 Autumn;34(3):328-36. <https://doi.org/10.1521/suli.34.3.328.42774>
