# Supplementary material for: A narrative review of the impact of the transition to ICD-10 and ICD-10-CM/PCS
Source: JAMIA Open. 2019 Dec 26;3(1):126–31. doi: 10.1093/jamiaopen/ooz066 (PMC7309233; doi:10.1093/jamiaopen/ooz066)
Supplement: ooz066_Supplementary_Data [file ooz066_supplementary_data.zip › Supplementary File 3_2019-10-21-clean.docx]

**SUPPLEMENTARY FILE 3: Additional Studies Addressing Other Concerns**

**Contents**

[Staffing Plans 1](#_Toc16257336)

[Impact of Computer-Assisted Coding (CAC) 1](#_Toc16257337)

[System Readiness 2](#_Toc16257338)

[System Testing 2](#_Toc16257339)

[Timing of Training 3](#_Toc16257340)

[Other Training Concerns 4](#_Toc16257341)

[Other Mapping Concerns 5](#_Toc16257342)

[References 6](#_Toc16257343)

### **Staffing Plans**

Prior to implementation, Houser and colleagues (2013) surveyed health information management/health administrative personnel from Alabama hospitals on their plans for the transition.[1] The survey was conducted from December 2011 through February 2012 and found that, of 43 responding organizations, including governmental, non-profit, and for-profit hospitals, 35% planned to hire additional coders and 18% planned to increase hours for coding staff to account for anticipated productivity losses following implementation.

### **Impact of Computer-Assisted Coding (CAC)**

Many organizations implemented computer-assisted coding (CAC) applications around the same time as the transition to ICD-10.[2] This may have had both positive and negative impacts on productivity in the ICD-10 environment that are difficult to fully assess. For example, Rudman et al. (2016) reported a coding environment-mediated negative impact of CAC use on productivity based on survey results from 156 coding professionals, with individuals using CAC experiencing an average productivity decrease of 17.13 percent, compared with an average decrease of 11.92 percent for those not using CAC.[3] The majority of respondents reporting CAC use worked in inpatient settings, which had a slightly higher perceived decrease in productivity (24.30% inpatient decrease, 22.10% outpatient decrease). The authors report, “When controlling for setting (in-patient/outpatient), differences do not exist in rates in the use of CAC when coding records (-16.73% and -17.19%, respectively).” Additionally, accuracy increased for those using CAC by 0.2%, compared with a decrease in accuracy of 1.58% for those not using CAC. The authors recommended conducting additional research to examine the interaction between ICD-10 implementation and CAC use, including coder familiarity with the CAC technology.

On the other hand, CAC is generally perceived and marketed as a productivity enhancement. The AHIMA Foundation and Cleveland Clinic, with funding from 3M, studied the impact of CAC on coder time and data quality for ICD-9 coding.[4] Twelve coders were divided into parallel study arms of credentialed coder and CAC versus credentialed coder alone and assigned ICD-9-CM codes to 25 patient cases within weeks of implementing the CAC and at six months post-implementation. Cases were complex; average case mix index was 2.45, and average length of stay was 16 days. Using a CAC reduced coder time per record by 22% and did not decrease accuracy. This study also reported an increase in precision and recall of the CAC’s natural language processing algorithm at the six-month mark. Vendors similarly report inpatient coder productivity increases of up to 25% in their marketing materials (3M, 2019; Dolbey, 2018; Optum, 2018).[5-7]

### **System Readiness**

A report by the Workgroup for Electronic Data Interchange (WEDI) ICD-10 Workgroup Testing Subworkgroup of lessons learned for specific stakeholder populations from the ICD-10-CM/PCS transition indicated that, “For providers that rely on software vendors, some did not receive system updates until late in 2015, or were unaware that system updates were necessary.”[8]

A March 2016 WEDI survey of 66 individuals reported that a “small number” of provider organizations indicated that the electronic health record or practice management system in use at their organization was not ICD-10 ready by the October 1, 2015, implementation date.[9]

Monestime and colleagues (2019) describe system readiness issues and their ramifications at a single outpatient public health institution in Florida. Semi-structured interviews with coders, physicians, department heads, and managers were conducted in October 2015, June 2016, and May 2018.[10] All participants indicated that the institution’s EHR system was not ready at the time of ICD-10-CM implementation. The system was expected to be ready in March 2016, and providers were told to continue using ICD-9-CM codes until the system updates were completed.[10] This led to difficulties with selecting ICD-10-CM codes when clinical documentation was in ICD-9-CM (reported by 5 of 9 participants). The system was not fully complaint until June 2016, and the delay in software readiness resulted in the need for retraining physicians in ICD-10-CM.

### **System Testing**

One organization of the Cooperative Exchange ICD-10 Work Group reported that their practice management software was not ready for ICD-10; however, through testing with payers, they were able to “identify payer issues prior to go live where one issue not being caught before go live could have caused millions of claims to be in error.”[11]

A December 2015 Navicure survey of 360 individuals from U.S. physician practices reported that 51% participated in end-to-end testing, which was described as somewhat or extremely helpful by 82% of those who participated.[12]

Finding the right contacts at a health plan to initiate testing was highlighted as an issue for some providers and clearinghouses. ICD-10 testing requirements were limited by differing specifications among payers, sometimes vague test cases, and frequent requirements changes. Testing environments were often not provided, so much of the testing for ICD-10 readiness occurred in production environments or manually, such as in spreadsheets or with paper responses from the payer. Testing in production created some confusion due to highly similar actual Remittance Advices and testing Remittance Advices. While core systems were often ready for ICD-10, ancillary systems and reports were overlooked, leading to some problems discovered just before implementation.[8]

### **Timing of Training**

In April 2016, the Workgroup for Electronic Data Interchange (WEDI) ICD-10 Workgroup Testing Subworkgroup published a set of lessons learned from the ICD-10 transition to “serve as a starting point for future projects that are similar in scope to the ICD-10 migration.”[8] Among their recommendations, the Testing Subworkgroup identified several areas of difficulty in ICD-10 testing. They reported that, “Some providers waited too long to get their staff trained for ICD-10,” including many smaller providers, which led to delays in other tasks, including testing.[8]

Monestime and colleagues (2019) conducted interviews and observations with “13 department heads, managers, physicians, and coders in a single outpatient public health institution in Florida” over the three-year period following ICD-10 implementation.[10] Interviews, observations, and “mini chart reviews” were conducted during the ICD-10 implementation period in October 2015 (n = 9), with follow-ups conducted in June 2016 (telephone interviews with 3 original participants) and in May 2018 (in-person interviews with 4 original participants).[10] Nine participants were interviewed about the go-live period, and 11 were interviewed about the period following implementation. All 9 respondents for the go-live period indicated “coder and physician ICD-10-CM training was a critical approach for successful implementation,” but five respondents stated that “training was rushed, and more time should have been allotted.”[10] Additionally, 56% (6/11) of post-implementation respondents reported physicians “struggled with selecting ICD-10-CM codes and relied more on the coders to choose the correct codes” 7 months after go-live, attributed to a “delay in system readiness,” and therefore physicians had to be retrained in ICD-10 coding.[10]

In a survey conducted as part of the 2003 American Hospital Association (AHA) and American Health Information Management Association (AHIMA) ICD-10-CM field testing with 169 health information management professionals, the majority of respondents (58.6%) indicated that ICD-10-CM training should be given three months ahead of implementation, while 29% preferred a timeframe of 6 months before implementation, and 9% selected 1 year before implementation.[13]

In an article describing the implementation of the Australian modification of ICD-10 (ICD-10-AM) in Australia, Innes and colleagues (2000) report, “The timing of release of the first edition—to be ready for the education and preparation of coders, data users, and hospital systems—was a critical issue.[14] The two years from the decision to change to actual implementation was insufficient lead time to build the classification and educate users.”[14]

### **Other Training Concerns**

A 2016 presentation by Romano on opportunities and challenges of the ICD-10 implementation notes that the dual coding instituted at the Washington State Department of Health (8 hospitals) and at the University of California Davis Medical Center (6 and 12 months prior to the transition, respectively) aided in staff training.[15]

In an article addressing pre-implementation planning, productivity and training issues to minimize transition impact, Rahmathulla and colleagues (2014) suggest enlisting physician champions, especially for training other physicians about what they need to know vis-à-vis the new coding systems (e.g., changes to point of care systems, reinforcing the need to adequately detail patient encounters to provide coders with sufficiently specific information).[16] Physician champion trainers/consultants can provide support that will reduce loss of productivity by both physicians and coders.

Sand and Elison-Bowers (2013) sought to determine whether individuals with experience in ICD-9-CM coding could transfer knowledge to ICD-10-CM/PCS.[17] Using a knowledge questionnaire, the investigators assessed the performance of participants with no ICD-9-CM knowledge (Group 1; n = 11) in comparison with experienced ICD-9-CM coders (Group 2; n = 20). Neither group had received formal ICD-10 training. Mean scores were calculated for each group and an analysis was conducted using an independent-samples t-test. The study found that, while “respondents in Group 1 had significantly lower ICD-10-CM subscale scores (*M* = 4.0909) than respondents in Group 2 (*M* = 6.1000), *t*(29) = −2.009, *p* = .002,” the knowledge transfer did not appear to extend to ICD-10-PCS, as “no statistical significance was found in ICD-10-PCS subscale scores between Group 1 (*M* = 4.000) and Group 2 (*M* = 4.7000), *t*(29) = −.7, *p* = .304.”[17] The authors suggested that these findings indicated even experienced coders required training for ICD-10/CM/PCS but could potentially need less overall training time due to their knowledge of ICD-9-CM.[17]

Houser et al. (2013) published results of a survey from the Alabama Association of Health Information Management directory on readiness, training approaches, and challenges associated with ICD-10 preparation.[1] The survey was conducted in December 2011, and follow-ups were completed in February 2012. Ninety-three percent of the 43 respondents were “HIM directors or managers,” with the remaining 7% categorized as “hospital administrative personnel.”[1] Of the 41 participants with valid responses, 41% (n = 17) were affiliated with a federal or nonfederal government hospital, 36.6% (n = 15) with a nonprofit hospital, and 22% (n = 9) with an investor-owned for-profit hospital. Training strategies offered at the respondents’ institutions were in-house training (58%), seminars/webinar for employees (58%), consultant-provided courses (33%), and seminars/webinars for coders (18%), coder-specific courses (9%). Three percent of respondents indicated no known plan for training at their institutions.[1]

In the 2003 AHA/AHIMA ICD-10-CM field testing survey, the majority of the 169 respondents (60%) indicated “16 hours or less” of training was needed prior to implementation, while 24.1% selected that “17 to 24 hours” was needed, and 11.7% felt that “25 to 32 hours” was needed.[13] Face-to-face training was the preferred method of delivery for the majority of respondents (76.6%), with “Internet-based training” selected by 47.6% of respondents as the most popular second choice.[13]

### **Other Mapping Concerns**

Wang and colleagues (2013) reported results from a survey conducted by the New York State Congenital Malformations Registry (CMR) of 91 hospitals in September-October 2012.[18] Although the survey was focused on the hospitals’ readiness for reporting data to the CMR, respondents also reported the methodology used at their organization for converting from ICD-9-CM to ICD-10-CM/PCS: “about 51% of the 91 respondents answered that they will rely on a crosswalk provided by a vendor, 7% will use the general equivalence mapping method…, 10% use other methods (ICD-9-CM and ICD-10-CM/PCS coding books), and 33% were not sure.”[18] This survey was conducted prior to the delays of ICD-10 implementation until October 2015; responses may not indicate final mapping methods used by these institutions.

CMS transitioned the Inpatient Rehabilitation Facility Prospective Payment System (IRF PPS) from ICD-9-CM to ICD-10-CM in Fiscal Year 2015 via a forward mapping through the General Equivalence Mappings (GEMs). ICD-10-CM codes are used to determine eligibility of a facility to be paid through IRF PPS, via annual presumptive compliance. Presumptive compliance computerized algorithms automatically classify the primary reason the patient is being treated, as ICD-10-CM codes, into 13 impairment group codes (IGCs), where at least 60% of a facility’s inpatient population must be in an IGC. The set of codes defining each IGC was not reviewed for clinical relevance at that time of transition to ICD-10-CM, and was instead clinically reviewed as part of the Final Rule 82 FR 36238 in 2017. The authors reported, “Our comprehensive review of the ICD-10-CM code lists for the presumptive methodology showed that excluded diagnosis codes listed in two IGC categories were affected by the ICD-10-CM translation: Traumatic brain injury (TBI) and hip fracture(s).”[19] Further, ICD-9-CM codes for patients with major multiple trauma were incorrectly mapped to a single unspecified ICD-10-CM code where multiple specific ICD-10-CM codes are more appropriate. Unspecified ICD-10-CM codes related to arthritis and myopathies were considered for removal due to some facilities’ disproportionate coding where more specific codes would have been appropriate. However, these changes were not finalized to allow use in the uncommon instances where greater detail is not available. Overall, the Final Rule 82 FR 36238 changes to IGCs revealed areas where a simple mapping using the GEMs was insufficient.

### **References**

1. Houser SH, Morgan D, Clements K, et al. Assessing the planning and implementation strategies for the ICD-10-CM/PCS coding transition in Alabama hospitals. *Perspect Health Inf Manag* 2013;10:1a.
2. Eramo LA. Has CAC lived up to its promise? Providers, vendors weigh in. *J AHIMA* 2017 Jun 1. <https://journal.ahima.org/2017/06/01/has-cac-lived-up-to-its-promise-providers-vendors-weigh-in/> Accessed October 17, 2019.
3. Rudman, WJ, Jackson K, Shank P, et al. Perceived effects of ICD-10 coding productivity and accuracy among coding professionals. *Perspectives in Health Information Management* 2016:1-10. <https://perspectives.ahima.org/perceived-effects-of-icd-10-coding-productivity-and-accuracy-among-coding-professionals/> Accessed October 17, 2019.
4. Dougherty M, Seabold S, White SE. Study reveals hard facts on CAC. *J AHIMA* 2013;84(no. 7):54-56. <http://library.ahima.org/doc?oid=106668#.XN3GRPZFxPY> Accessed October 17, 2019.
5. 3M. 3M 360 Encompass System. St. Paul (MN): 3M; 2019 <https://www.3m.com/3M/en_US/360-encompass-system-us/computer-assisted-coding/> Accessed October 17, 2019.
6. Dolbey Systems. Computer-assisted coding solutions. Concord (OH): Dolbey Systems, Inc.; 2018. <https://www.dolbey.com/wp-content/uploads/documentation/dolbey-computer-assisted-coding.pdf>

Accessed October 17, 2019.

1. Optum. Success snapshot. 2018. <https://www.optum360.com/content/dam/Optum360/Resources/articles/SS-HAP-CACPro-billing-2018.pdf> Accessed October 17, 2019.
2. Workgroup for Electronic Data Interchange (WEDI) Strategic National Implementation Process ICD-10 Workgroup - Testing Subworkgroup. Testing: lessons learned from ICD-10. Reston (VA): WEDI; 2016 Apr 29 <https://www.wedi.org/knowledge-center/resource-view/resources/2016/04/29/testing---lessons-learned-from-icd-10> Accessed April 11, 2019.

1. Workgroup for Electronic Data Interchange (WEDI). WEDI ICD-10 post-implementation survey results released. 2016 May 9. <https://www.wedi.org/news/press-releases/2016/05/09/wedi-icd-10-post-implementation-survey-results-released> Accessed April 30, 2019.
2. Monestime JP, Mayer RW, Blackwood A. Analyzing the ICD-10-CM Transition and Post-implementation Stages: A Public Health Institution Case Study. *Perspect Health Inf Manag* 2019;16(Spring):1a.
3. Gomez B, Kossow J. Clearinghouse Realities of ICD-10. Cooperative Exchange; 2015 Dec 10. <https://www.wedi.org/forms/uploadFiles/6F36E00000010.toc.WEDI_ICD-10_revised_final.pdf>

Accessed April 29, 2019.

1. Navicure. Healthcare organization post ICD-10 implementation survey: Key survey findings. 2016 Jan <https://web.archive.org/web/20180219123237/http:/info.navicure.com/rs/669-OIJ-380/images/Navicure-Post-ICD-10-Survey_Final.pdf> Accessed October 17, 2019.
2. American Hospital Association (AHA) and American Health Information Management Association (AHIMA). ICD-10-CM field testing project: report on findings: perceptions, ideas and recommendations from coding professionals across the nation. Washington, D.C.; The Associations; 2003 Sep 23 <http://library.ahima.org/doc?oid=61292#.XREsu49OmUm> Accessed October 17, 2019.
3. Innes K, Peasley K, Roberts R. Ten down under: implementing ICD-10 in Australia. *J AHIMA* 2000;71(1):52-6. <http://library.ahima.org/doc?oid=57594#.XNrqg45KiCp> Accessed October 17, 2019.
4. Romano PS. ICD-10 implementation: opportunities and challenges for health data organizations [Internet]. NAHDO Annual Meeting. 2016 Oct 27 <https://www.hcup-us.ahrq.gov/datainnovations/ICD10Imp_Romano_NAHDO_October2016.pdf> Accessed October 17, 2019.
5. Rahmathulla G, Deen HG, Dokken JA, et al. Implementation and impact of ICD-10 (Part II). *Surg Neurol Int* 2014;5(Suppl 3):S192-8. doi: 10.4103/2152-7806.137182.
6. Sand JN, Elison-Bowers P. ICD-10-CM/PCS: transferring knowledge from ICD-9-CM. *Perspect Health Inf Manag* 2013;10:1g.
7. Wang Y, Tao Z, Fox D, et al. A survey on readiness and needs regarding the transition from ICD-9-CM to ICD-10-CM. *J Registry Manag* 2013;40(1):4-8. <http://www.ncra-usa.org/Portals/68/PDFs/JRMSpring2013_40.01.pdf?ver=2017-08-03-121036-773> Accessed October 17, 2019.
8. Federal Register. Medicare program; Inpatient Rehabilitation Facility Prospective Payment System for federal fiscal year 2018. 2017 Aug 3;82(148):36238-36305. <https://www.govinfo.gov/content/pkg/FR-2017-08-03/pdf/2017-16291.pdf> Accessed October 17, 2019.
